# Identification and validation of novel platelet-related genes in ulcerative colitis using bioinformatics and machine learning strategies

**DOI:** 10.7717/peerj.21655

**Published:** 2026-08-25

**Authors:** Jin Huang, Ying Dai, Shuangshuang Lu, Xue Li, Ruihong Sun, Ben Huang, Chunmei Ma

**Affiliations:** 1 The Second People’s Hospital of Changzhou, Changzhou Medical Center, The Third Affiliated Hospital of Nanjing Medical University, Changzhou, China; 2 Department of Immunology, Nanjing Medical University, Nanjing, China; 3 Department of Laboratory Medicine, The First Affiliated Hospital of Nanjing Medical University, Nanjing, China; 4 The First School of Clinical Medicine, Nanjing Medical University, Nanjing, China

**Keywords:** Ulcerative colitis, Platelet-related genes, WGCNA, Immune cell infiltration, Machine learning, Single cell analysis

## Abstract

Ulcerative colitis (UC) is a chronic, relapsing inflammatory disorder of the colonic mucosa. Although colonoscopy remains the diagnostic cornerstone for UC, its invasive nature highlights the need for additional biomarkers. This study used differential expression analysis, weighted gene co-expression network analysis (WGCNA), and machine-learning algorithms to identify three key platelet-related genes (PRGs)—TRIM22, TIMP1, and RAC2—that are involved in UC development. Immune cell analysis and single-cell analyses indicated that the expression of these key genes was significantly correlated with macrophages, neutrophils, and mast cells. Moreover, these key PRGs were mainly involved in pathways related to UC, such as chemokine signaling and leukocyte transendothelial migration. The expression levels of the key PRGs were further validated in independent datasets, dextran sulfate sodium (DSS)-induced colitis tissues, and peripheral blood mononuclear cells (PBMC) samples from UC patients. Collectively, these findings support a platelet-related three-gene signature with potential diagnostic and biological relevance in UC.

## Key messages

**1. What is already known?**

Emerging evidence underscores the critical role of platelets in autoimmunity and inflammatory diseases, and platelet-related genes (PRGs) can help predict the prognosis of patients with triple-negative breast cancer and rheumatoid arthritis.

**2. What is new in present study?**

Three key platelet-related genes (PRGs)—*TRIM22*, *TIMP1*, and *RAC2*—were jointly prioritized as candidate biomarker signature in ulcerative colitis (UC) through weighted gene co-expression network analysis (WGCNA) and machine learning.

**3. How can this study help patient care?**

This study established a platelet-related three-gene signature associated with UC-related immune features and validated across multiple biological contexts, supporting its potential biomarker value in UC.

## Introduction

Global studies have demonstrated a rising incidence of ulcerative colitis (UC), imposing a considerable health burden (Du & Ha, 2020; Segal, LeBlanc & Hart, 2021). UC is a chronic immune-mediated intestinal pathology that predominantly affects the rectum and colon and can manifest at any age. UC patients suffer from chronic or intermittent diarrhea and mucopurulent and bloody stools, concomitant with abdominal discomfort, tenesmus, and other symptoms. The diagnosis of UC is often based on the patients’ clinical manifestations combined with endoscopic evaluation and histological parameters. While laboratory and radiographic tests can help diagnose UC, endoscopic investigation remains the diagnostic cornerstone (Kaenkumchorn & Wahbeh, 2020). Although colonoscopy is an effective strategy, it is an invasive operation, that can cause intestinal injury and inflammation, especially in patients with recurrent disease, making it unsuitable for frequent use for assessing the patient’s condition. In addition, patients with UC vary in severity, and patients in early stages may lack typical intestinal manifestations, which makes clinical diagnosis difficult. In clinics, the goals of treatment are to induce and maintain symptom relief and mucosal healing, prevent complications, and improve the quality of life of patients. Despite advanced therapeutics, approximately 10% to 20% of UC cases still necessitate proctocolectomy (Le Berre, Honap & Peyrin-Biroulet, 2023). Recent research suggests that integrative precision and personalized medicine might accelerate therapeutic advancements (Le Berre, Honap & Peyrin-Biroulet, 2023). Therefore, identifying potential biomarkers for UC is imperative for guiding clinical management.

Previous studies have revealed the potential role of platelets in UC. A clinical study reported significantly elevated platelet levels in UC patients, indicating platelets involvement in UC-associated inflammation (Chen et al., 2021). In addition, accumulating evidence suggests that UC is accompanied not only by thrombocytosis and altered platelet indices, but also by enhanced platelet activation, inflammatory mediator release, and platelet–leukocyte interactions (Pamuk et al., 2006; Sano et al., 2024). Platelets play important immunomodulatory roles in autoimmune and inflammatory diseases (Liu et al., 2023; Yazici et al., 2010; Scherlinger et al., 2017; Cortez-Espinosa et al., 2017; Dudiki et al., 2023). Platelet-related genes (PRGs) have also attracted increasing attention in human disease research. In the present study, PRGs were considered a biologically informed candidate space. Because platelets contribute to inflammation, immune regulation, tissue repair, angiogenesis, and thromboinflammatory responses in UC, systematic analysis of PRGs may help identify UC-relevant molecular signatures. Previous studies have shown that PRGs can contribute to disease diagnosis, prognosis, and immune-context interpretation in disorders such as rheumatoid arthritis and cancer (Xie et al., 2021). Nonetheless, their integrated roles in UC remain insufficiently characterized. Based on these findings, we hypothesized that a PRG-centered analytical framework could help identify candidate biomarkers and reveal immune-associated molecular features in UC.

The present study investigated platelet-related molecular features and immunological characteristics of UC by integrating six bulk Gene Expression Omnibus (GEO) cohorts and one single-cell RNA-sequencing dataset from GEO database. The GSE11223 dataset, which included 73 healthy controls and 129 UC samples, was used as the discovery and model-training cohort, whereas the remaining bulk datasets were used for external validation. Differential PRG expression, inferred immune-cell-fraction patterns, PRG-based subtype heterogeneity, and cell-type-specific expression landscapes were further explored. By integrating WGCNA and machine-learning approaches, we identified a three-gene signature consisting of TRIM22, TIMP1, and RAC2. We then interpreted these genes in the context of platelet-related molecular features, immune infiltration, and single-cell localization, and further validated them using a dextran sulfate sodium (DSS)-induced colitis mouse model and PBMC samples from UC patients as independent biological validation settings. Our results provide a PRG-centered framework for understanding UC biology and nominate candidate biomarkers for future investigation.

## Materials and Methods

### Data collection and processing

The relevant ulcerative colitis-related datasets were acquired from the Gene Expression Omnibus (GEO) database (https://www.ncbi.nlm.nih.gov/geo/). Six datasets were analyzed as bulk transcriptomic cohorts, including tissue cohorts (GSE11223, GSE107499, and GSE92415) and peripheral blood cohorts (GSE119600, GSE3365, and GSE94648). In addition, GSE214695 was analyzed as a single-cell RNA-sequencing dataset for cell-type-specific expression analysis. Details of the sample information were shown in Table S1. In our analysis, the six bulk GEO datasets were not merged into a single cross-platform matrix for joint discovery analysis; instead, each bulk cohort was processed independently. GSE11223 served as the discovery and model-training cohort, whereas the remaining bulk datasets were used as independent validation cohorts. The workflow of this study was shown in Fig. S1.

It is worth noting that the bulk expression data analyzed in this study were GEO-provided processed Series Matrix Files rather than raw sequencing reads or raw array image files. These processed matrices had already undergone platform-standard background correction during data generation, Reference genome and annotation information therefore followed the official GEO platform (GPL) platform annotation associated with each dataset, and no *de novo* alignment was performed in our workflow. Where applicable, probe identifiers in the Series Matrix File were matched to the corresponding GPL annotation file to obtain gene symbols, and when multiple probes mapped to the same gene, the mean expression value was used. The original gene expression matrix was processed using R version 4.4.2. Based on the sample lists of the control and UC groups, the corresponding expression data were extracted, log2-transformed, and then normalized across samples using the “normalizeBetweenArrays” function from the “limma” package.

#### Selection of platelet-related genes (PRGs)

The platelet-related genes (PRGs) were collected from platelet-related gene sets in Gene Set Enrichment Analysis/Molecular Signatures Database (GSEA/MSigDB) and from published literature describing experimentally validated platelet-function-related genes. A total of 485 platelet-related genes were obtained from these sources (Table S2).

In the present study, PRGs were defined as platelet-related biological candidate genes functionally linked to megakaryocyte/platelet biology, including platelet biogenesis, activation, adhesion, aggregation, secretion, thrombosis, platelet–leukocyte interaction, and thromboinflammatory processes, rather than as UC-specific genes or strictly platelet-specific transcripts. The rationale for introducing PRGs into the UC framework is not that all 485 genes are already known to be directly involved in UC, but that UC itself is increasingly recognized as a platelet-associated inflammatory disorder. Against this background, a PRG-centered candidate space provides a biologically meaningful starting point for identifying the subset of genes most relevant to UC.

### Identification of DEGs and functional enrichment analysis

Differential expression analysis was performed in R 4.4.2 using the processed GEO expression matrices. Differential expression was assessed using the Wilcoxon rank-sum test on a gene-by-gene basis, and genes with *P* < 0.001 were retained as difference expression genes (DEGs) for downstream analyses. Heatmaps were generated using the “pheatmap” package, and boxplots were generated using “ggpubr” and “ggplot2”. Subsequently, based on the “clusterProfiler” package, Gene Ontology (GO) and Kyoto Encyclopedia of Genes and Genomes (KEGG) analyses were performed to explore the molecular functions of the differential PRGs.

### Analysis of immune cell infiltration

This study used the CIBERSORT algorithm (Newman et al., 2015) to estimate the relative fractions of 22 immune cell types in each sample of the GSE11223 dataset. Because CIBERSORT estimates relative immune-cell fractions within each sample, the resulting infiltration profiles are compositional in nature and were interpreted as exploratory differences in relative immune composition rather than absolute immune-cell abundance. The LM22 reference gene set was used as the signature matrix for deconvolution analysis of the normalized expression matrix (Wang et al., 2023). The number of permutations was set to 1,000, and samples with a permutation-derived *P* value <0.05 were retained. The “e1071”, “preprocessCore”, and “parallel” packages were used for support vector regression, quantile normalization, and parallel computing, respectively. In addition, the relationships between PRGs and inferred infiltrating immune-cell fractions were explored using Spearman correlation analysis. The “corrplot” and “ggplot2” packages were used for visualization.

### Unsupervised cluster and gene set variation analysis (GSVA)

To explore the unidentified subtypes in UC, an unsupervised cluster analysis was performed based on the differentially expressed PRGs in the GSE11223 dataset. This analysis was intended as a PRG-centered subtype stratification rather than a whole-transcriptome molecular taxonomy of UC. The “ConsensusClusterPlus” package was used, and the consensus clustering parameter maxK was set to 9. A total of 129 UC samples were divided into different clusters using the k-means algorithm with 1,000 iterations. The optimal number of clusters was determined based on the empirical cumulative distribution function (CDF) of the consensus index across different K values, the corresponding delta area plot, consistency matrices, and clustering scores. Subsequently, GSVA was performed to compare pathway-level variation between the PRG-based UC subgroups. The “GSVA” and “GSEABase” packages were used, and the c2.cp.kegg.v7.4.symbols.gmt and c5.all.v7.0.symbols.gmt gene sets from MSigDB were selected as the reference gene sets. The gsva function with method = “gsva” was applied to calculate pathway enrichment scores for each sample, and the resulting scores were normalized to the [0, 1] range using min-max normalization. Based on the unsupervised clustering results (two clusters: C1 and C2), an independent two-sample t-test was performed for each pathway between the C1 and C2 groups. Pathways with *P* value <0.05 were considered significantly different. The top 10 upregulated and 10 downregulated pathways ranked by t value were selected, and a bar plot was generated using “ggpubr” package.

### Weighted gene co-expression network analysis (WGCNA)

WGCNA is an excellent algorithm for analyzing gene expression patterns in multiple samples, which can cluster genes with similar expression pattern. The “WGCNA” package was used for weighted gene co-expression network analysis. The pickSoftThreshold function was used to select the optimal soft threshold within the power index range of 1 to 20, targeting a scale-free topological fitting index R^2^ ≥ 0.90. Average-linkage hierarchical clustering was performed based on the topological overlap matrix (TOM), and the dynamic tree cut algorithm (deepSplit = 2, minimum module size = 100 genes) was used to identify gene modules. Pearson correlation analysis was used to calculate the correlation coefficients and *P* values between the module eigengenes and clinical traits. In this study, two rounds of WGCNA were performed to identify the key genes associated with UC. First, disease-related WGCNA was used to identify module signature genes significantly associated with disease status. Second, cluster-related WGCNA was performed to identify module signature genes significantly associated with the PRG-based subgroups. The intersection of the signature genes identified by the two WGCNA analyses was taken as the final candidate gene set for machine-learning analysis.

### Identification of key PRGs by machine learning algorithms

In this study, the discovery and model-training cohort (GSE11223; used for selecting key genes) was randomly divided into a training set and a fixed test set at a 7:3 ratio using the caret package, and the other datasets were used for independent validation. The machine-learning analysis was designed as an exploratory feature-prioritization step. Four machine-learning models, including random forest (RF), support vector machine (SVM), eXtreme Gradient Boosting (XGB), and generalized linear models (GLM), were constructed for candidate-gene prioritization (Kuhn, 2008; Liaw & Wiener, 2002; Karatzoglou et al., 2004). R packages “caret”, “randomForest”, “kernlab”, “xgboost”, and “DALEX” were utilized (Kuhn, 2008; Liaw & Wiener, 2002; Karatzoglou et al., 2004; Chen & Guestrin, 2016; Biecek, 2018). Model training and hyperparameter tuning were performed using five-fold repeated cross-validation within the training set. RF was implemented with method = “rf”; SVM with method = “svmRadial” and prob.model = TRUE; XGB with method = “xgbDART”; and GLM with method = “glm” and family = “binomial”. All other parameters for each model were kept at the default values of the caret package.

After model training, the fixed test set was used to plot receiver operating characteristic (ROC) curves and compare the predictive performance of the four models using the “pROC” package. The ggplot2 package was used to generate residual reverse cumulative distribution plots and residual boxplots. The DALEX package was used to explain model performance (assessing gene importance based on the root mean square error loss function) and to output the genes with the highest importance scores. The top three genes from the best-performing model were subsequently selected as the key PRGs for downstream analyses, whereas the remaining UC tissue and blood datasets were used for independent validation rather than model training.

### Single gene GSEA analysis

To further understand the biological pathways associated with the key genes in UC, gene set enrichment analysis (GSEA) was performed using the “clusterProfiler”, “enrichplot”, and “org.Hs.eg.db” packages. For each signature gene, samples in the GSE11223 cohort were divided into high-expression and low-expression groups according to the median expression level of the corresponding gene. The log2 fold change (log2FC) for each gene across the whole transcriptome was then calculated between the high- and low-expression groups, and all genes were ranked in descending order according to their log2FC values. The full ranked gene list was used as input for GSEA using the KEGG gene set (c2.cp.kegg.v7.4.symbols.gmt), and pathways with *P* < 0.05 were considered significantly enriched. The corresponding R script was provided as Supplemental Material.

### Single cell analysis

To further explore the expression of the signature genes at the single-cell level in UC, we downloaded GSE214695 from GEO for single-cell analysis. This dataset included six healthy control samples and six UC samples. The processed Seurat object was analyzed using Seurat, and the metadata fields nanostring_reference and sample_group were used as the primary cell-type and disease-group annotations for downstream analyses. Thus, the existing annotation provided in the processed dataset was used as the main cell-type labeling framework for this study, rather than performing a fully *de novo* global re-annotation or *de novo* alignment workflow. To support annotation plausibility, canonical marker genes from immune, epithelial, and stromal lineages were examined using FeaturePlot. Uniform Manifold Approximation and Projection (UMAP) embeddings stored in the processed object were used for visualization, and downstream analyses included overall UMAP display, healthy control/ulcerative colitis (HC/UC) split visualization, violin plots, split violin plots, dot plots, marker exploration, sample-level and cell-type-level proportion analyses, and marker summarization using FindAllMarkers. Harmony was used in the downstream workflow to reduce potential batch effects during single-cell analysis and visualization.

### Real-time quantitative reverse transcription PCR analysis

In this study, peripheral blood mononuclear cells (PBMCs) were extracted from the clinical UC patients’ blood samples by density gradient centrifugation, and then lysed with Trizol reagent (Thermo Fisher Scientific, Darmstadt, Germany). In the DSS model, colonic tissues were homogenized using Trizol reagent, and total RNA was subsequently extracted. RNA purity and concentration were assessed before reverse transcription. Reverse transcription and quantitative real-time polymerase chain reaction (PCR) were conducted in strict accordance with the protocols provided by Vazyme Biotechnology (Nanjing) Co., Ltd. β-actin was used as the internal reference for relative quantification. Quantitative reverse transcription (qRT)-PCR was conducted using a SYBR Green PCR Kit (Q121-02; Vazyme, Nanjing, China). Samples were analyzed in duplicate across all groups using an ABI 7500 Thermocycler (Applied Biosystems). The relative mRNA expression levels were quantified using the 2-ΔΔCt method. Primer sequences are listed in Table S3. This study was approved by the Ethics Committee of the Affiliated Hospital of Nanjing Medical University (2025-SR-1243) and in accordance with the principles of the Declaration of Helsinki. Written informed consent was obtained from all participants before sample collection. RNA concentration was measured using the Onedrop1000 instrument. Contamination assessment was performed based on spectrophotometric purity ratios. Samples with an A260/280 ratio between 1.8 and 2.0 were considered acceptable, whereas ratios <1.8 indicated potential protein contamination and ratios >2.0 suggested possible RNA degradation. The A260/230 ratio was expected to be around ~2.5; values <2.0 indicated possible contamination with carbohydrates, salts, or organic reagents (*e.g*., phenol or guanidine). Samples that did not meet these purity criteria were excluded from downstream experiments. RNA integrity was inferred from successful amplification and single-peak melt curves. Primers were checked using NCBI BLAST; only the target gene was identified. Outliers were excluded only if melt-curve abnormal or technical replicate variation was >0.5 Cq.

### Establishment of a DSS-induced colitis mouse model

For colitis induction, 8–9-week-old male C57BL/6 mice were treated with 2.5% DSS in drinking water for 6 days, followed by normal drinking water until the end of experiment on day 8. Mouse body weight, stool, and body posture were monitored daily to calculate the disease activity index (DAI). The DAI is the combined score of weight loss compared with initial weight, stool consistency and blood stool, and body posture. The scores were evaluated as follows: weight loss: 0 (no loss), 1 (5% to 10%), 2 (10% to 15%), and 3 (>15%); stool consistency: 0 (normal), 1 (mild loose stool), 2 (loose stool), and 3 (diarrhea and bloody stools); body posture: 0 (smooth fur without a hunchback), 1 (mild fur and hunchback), 2 (moderate fur and hunchback), and 3 (severe fur and heavy hunchback). Mice were euthanized at day 8, and the mouse colons were collected immediately for colon length measurement, qRT-PCR, western blot, and histology analysis. This study was approved by the Ethical Review Committee for Laboratory Animal Welfare of Nanjing Medical University (IACUC:2202013-3).

### Western blot analysis

Colonic tissue homogenates were lysed in lysis buffer solution supplemented with a protease inhibitor cocktail tablet (Sigma-Aldrich). Samples were clarified, denatured with SDS buffer, and boiled for 10 min. Proteins were separated by SDS–polyacrylamide gel electrophoresis and transferred onto nitrocellulose membranes. The membranes were incubated with primary antibodies at 4 °C, and then with appropriate secondary anti-rabbit antibody conjugated to HRP at room temperature for 1 h. The following primary antibodies were used: anti-TRIM22 (FNab08973, 1:500; Fntest, Wuhan, China), anti-RAC1/2 (CY6802, 1:1000; Abways, Shanghai, China), anti-TIMP1 (A1389, 1:1000; ABclonal, Woburn, MA, USA), and anti-β-actin (A1978, 1:2000; Sigma-Aldrich, Burlington, MA, USA). Immunoreactivity was detected using a chemiluminescence system.

### H&E staining and histological analysis

The mouse colons were washed with phosphate-buffered saline (PBS), fixed in 4% formaldehyde, embedded in paraffin, sectioned at 5 μm, and stained with hematoxylin and eosin (H&E). Histological injury was evaluated using a modified semi-quantitative scoring system based on previously described criteria for DSS-induced colitis. The scoring system included three pathological parameters: ulceration/erosion, epithelial damage, and inflammatory cell infiltration. Ulceration/erosion was scored according to the presence and number of ulcerative or erosive lesions. Epithelial damage was scored as follows: 0, normal mucosal architecture; 1, mild epithelial hyperplasia, irregular crypts, or goblet cell loss; 2, moderate crypt distortion, focal crypt loss, or focal epithelial erosion; 3, severe crypt loss, epithelial disruption, or focal ulceration. Inflammatory cell infiltration was scored as follows: 0, no obvious inflammatory infiltrate or only occasional inflammatory cells in the lamina propria; 1, mild inflammatory cell accumulation in the lamina propria; 2, moderate inflammatory infiltration extending into the mucosa and submucosa; 3, marked inflammatory infiltration involving the mucosa and submucosa, with occasional transmural extension; and 4, severe transmural inflammatory infiltration. The total histological score was calculated as the sum of the ulceration/erosion, epithelial damage, and inflammatory infiltration subscores, with a maximum possible score of 8. Higher scores indicated more severe colonic injury. Histological assessment was performed independently by Ying Dai and Chunmei Ma in a blinded manner. Periodic acid–Schiff (PAS) staining was performed to visualize goblet cells. Images were acquired using a Nikon 50i microscope.

### Statistical analysis

R 4.4.2 software was used for statistical analysis. The Wilcoxon test and t-test were used to compare differences in PRG expression between the healthy control group and the UC group. Spearman correlation analysis was used to assess the association between PRG expression and inferred immune-cell fractions estimated by CIBERSORT. ROC curves were used to evaluate the diagnostic value of the signature genes. *P* < 0.05 was considered statistically significant.

## Results

### Identification of platelet-related differential genes in UC

**Figure 1:**
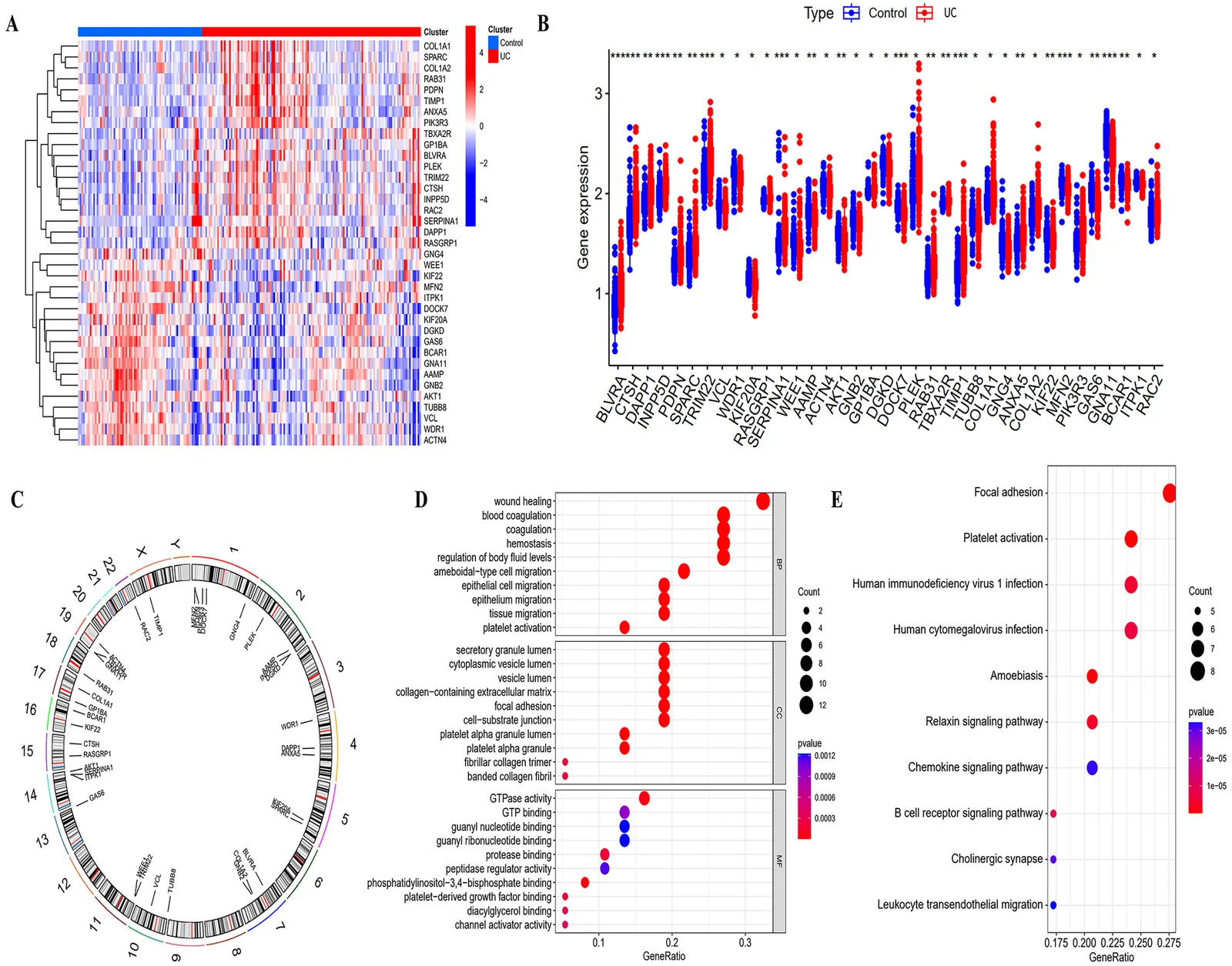
Identification of platelet-related differential genes in UC. (A) Red and blue colors represent genes with high and low expression, respectively, and 37 differential PRGs were finally identified. (B) 19 genes were up-regulated and 18 genes were down-regulated. (C) Location of 37 PRGs on chromosomes. (D) GO analysis of the 37 PRGs in UC. (E) KEGG analysis of the 37 PRGs in UC. Asterisks indicate statistical significance between the UC and control groups: *P < 0.05, **P < 0.01, ***P < 0.001, and ****P < 0.0001.

In the GSE11223 dataset, the expression levels of 485 PRGs were compared between the UC group and the healthy control group using the Wilcoxon test. At a *P* < 0.001, 37 differential PRGs were identified (Table S4), and the heatmap was shown in Fig. 1A. Of these 37 differential PRGs, 19 genes were upregulated and 18 genes were downregulated (Fig. 1B). The chromosomal locations of these DEGs were shown in Fig. 1C. GO enrichment analysis indicated that the 37 differential PRGs were mainly involved in biological processes such as wound healing, blood coagulation, and hemostasis, which are expected findings for a platelet-related gene set (Fig. 1D). Result of the KEGG analysis suggested that the 37 differential PRGs were involved in focal adhesion, platelet activation, and the chemokine and B-cell receptor signaling pathways (Fig. 1E).

### Immune cell infiltration analysis

The CIBERSORT algorithm was used to evaluate differences in the inferred relative fractions of 22 infiltrating immune cell types between the UC group and the healthy control group. The results revealed significant disparities in resting CD4 memory T cells, resting natural killer (NK) cells, resting dendritic cells, activated mast cells, and neutrophils between the groups (Figs. S2A, S2B). In addition, a Spearman correlation analysis demonstrated a complex relationship between the 37 differential PRGs and the inferred immune-cell fractions (Fig. S2C). For instance, *TRIM22* exhibited a substantial positive correlation with M0 and M1 macrophages (Figs. S2D, S2E) and a negative correlation with M2 macrophages and resting CD4 memory T cells (Figs. S2F, S2G). These findings suggest that PRG expression was associated with the immune context of UC.

### Unsupervised cluster analysis and GSVA analysis

**Figure 2:**
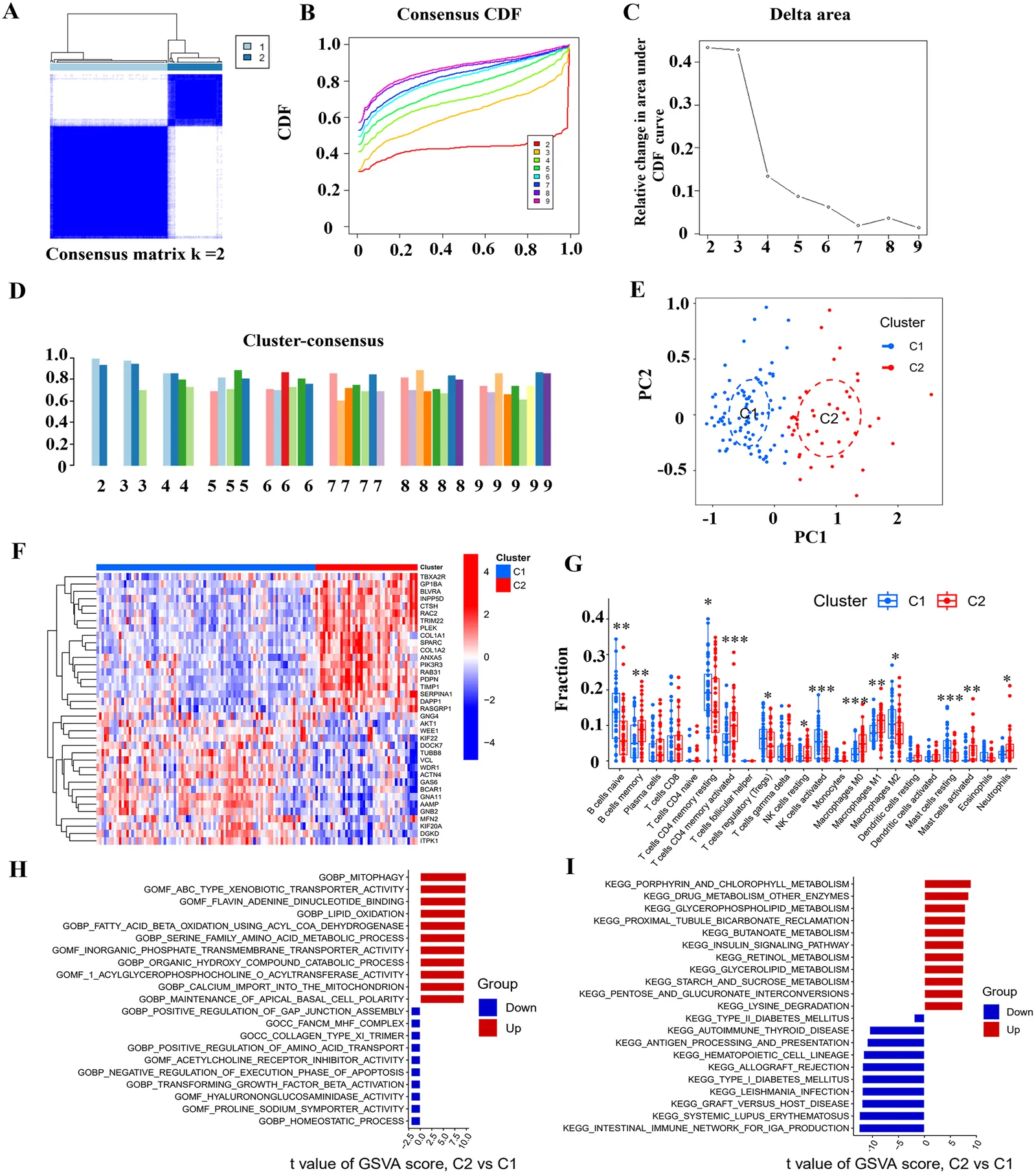
Unsupervised cluster analysis and GSVA analysis. (A) Consensus clustering matrix (k = 2). (B) The cumulative distribution function (CDF) curves. (C) CDF delta area curves. (D) Scores of consensus clustering. (E) PCA analysis. (F) Heatmap of PRGs in clusters. Red and blue colors represent genes with high and low expression, respectively. (G) Boxplots showed the differences of 22 infiltrated immune cells in clusters. (H) Differences in biological functions between cluster 1 and cluster 2. (I) Differences in pathway activities between cluster 1 and cluster 2. Asterisks indicate statistical significance between the two clusters: *P < 0.05, **P < 0.01, and ***P < 0.001.

In the GSE11223 dataset, an unsupervised cluster analysis was performed based on the expression of 37 differential PRGs to define PRG-based UC subgroups. Results showed that when K = 2, the subgroup separation and clustering stability were most appropriate (Fig. 2A). The cumulative CDF curves of the consensus index and the corresponding delta area plot were used to evaluate clustering stability across different K values (Figs. 2B, 2C). Increasing K beyond 2 provided limited improvement in clustering stability, supporting the selection of K = 2. Therefore, 129 UC samples were divided into two clusters, with 88 samples in cluster 1 (C1) and 41 samples in cluster 2 (C2). Results of the principal component analysis (PCA) also demonstrated clear separation between the two PRG-based subgroups (Figs. 2D, 2E).

The expression patterns of the 37 differential PRGs in the two PRG-based subgroups were shown in Fig. 2F. Differences in inferred immune-cell fractions between the two clusters were analyzed by CIBERSORT algorithm. Results showed significant differences in the naïve B cells, memory B cells, resting/activated memory CD4 T cells, NK cells, macrophages, mast cells, and neutrophils between the two clusters. Cluster 2 had a higher inferred fractions of memory B cells, M1 macrophages, neutrophils, and activated CD4 T cells, and lower inferred fractions of M2 macrophages (Fig. 2G), indicating substantial differences in immune context between the PRG-based subgroups.

Subsequently, differences in pathway activity between the PRG-based subgroups were further explored using GSVA. Results showed that positive regulation of gap junction assembly, regulation of amino acid transport, negative regulation of execution phase of apoptosis, and transforming growth factor beta activation were enriched in cluster 1, whereas sphingomyelin phosphodiesterase activity, mitophagy, serine-family amino acid metabolic process, and inorganic phosphate transmembrane transporter activity were enriched in cluster 2 (Fig. 2H). KEGG-related pathway enrichment further suggested that antigen processing and presentation, hematopoietic cell lineage, and the intestinal immune network for IgA production were enriched in cluster 1, whereas drug metabolism, glycerophospholipid metabolism, and insulin signaling pathways were enriched in cluster 2 (Fig. 2I).

### WGCNA results

**Figure 3:**
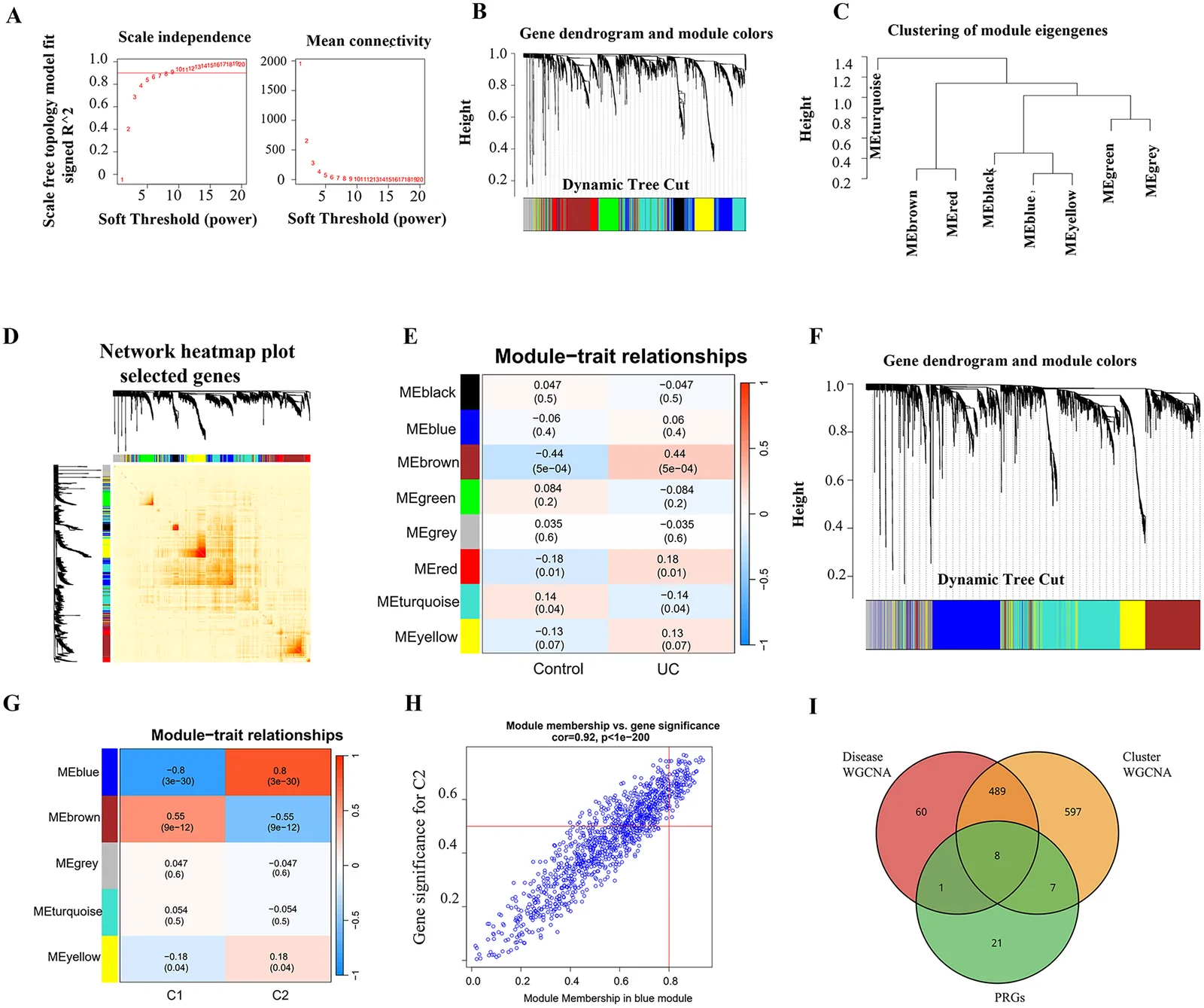
Results of WGCNA analysis. (A) Selection of soft threshold power and mean connectivity. (B) Cluster tree dendrogram of co-expression modules. Different modules were shown in different colors. (C) Clustering of module genes. (D) Heatmap of the correlations among modules. (E) Correlation analysis between module signature genes and clinical status. (F) Cluster tree dendrogram of co-expression modules. (G) Correlation analysis between module signature genes and clusters. (H) Correlation between module membership and the gene significance in blue module. (I) Venn diagram showed the intersecting genes.

In order to identify key gene modules associated with UC, co-expression networks and gene modules were constructed using the WGCNA algorithm. Co-expressed gene modules were identified at a scale-free R^2^ of 0.9 and a soft thresholding power of 7 (Fig. 3A). The dynamic tree cut method (Figs. 3B) was used to delineate eight distinct modules (Figs. 3C). These modules were coded for differentiation, and presented in a heatmap of their topological overlap matrix (Figs. 3D). These modules were further analyzed to determine the similarity and association of their gene co-expression patterns with clinical features of UC. Notably, the brown module, composed of 558 genes, demonstrated a significant correlation with UC (Fig. 3E).

In a parallel analysis for clusters using WGCNA, the blue module emerged as being significantly related to clusters (Figs. 3F, 3G). A correlation analysis substantiated a significant association between the genes of the blue module and those selected from the modules (Fig. 3H). Based on these results, a Venn diagram analysis identified eight overlapping genes between the differential PRGs and gene modules, offering a foundation for developing machine learning algorithms (Fig. 3I).

### Identification of key PRGs by machine learning algorithms

**Figure 4:**
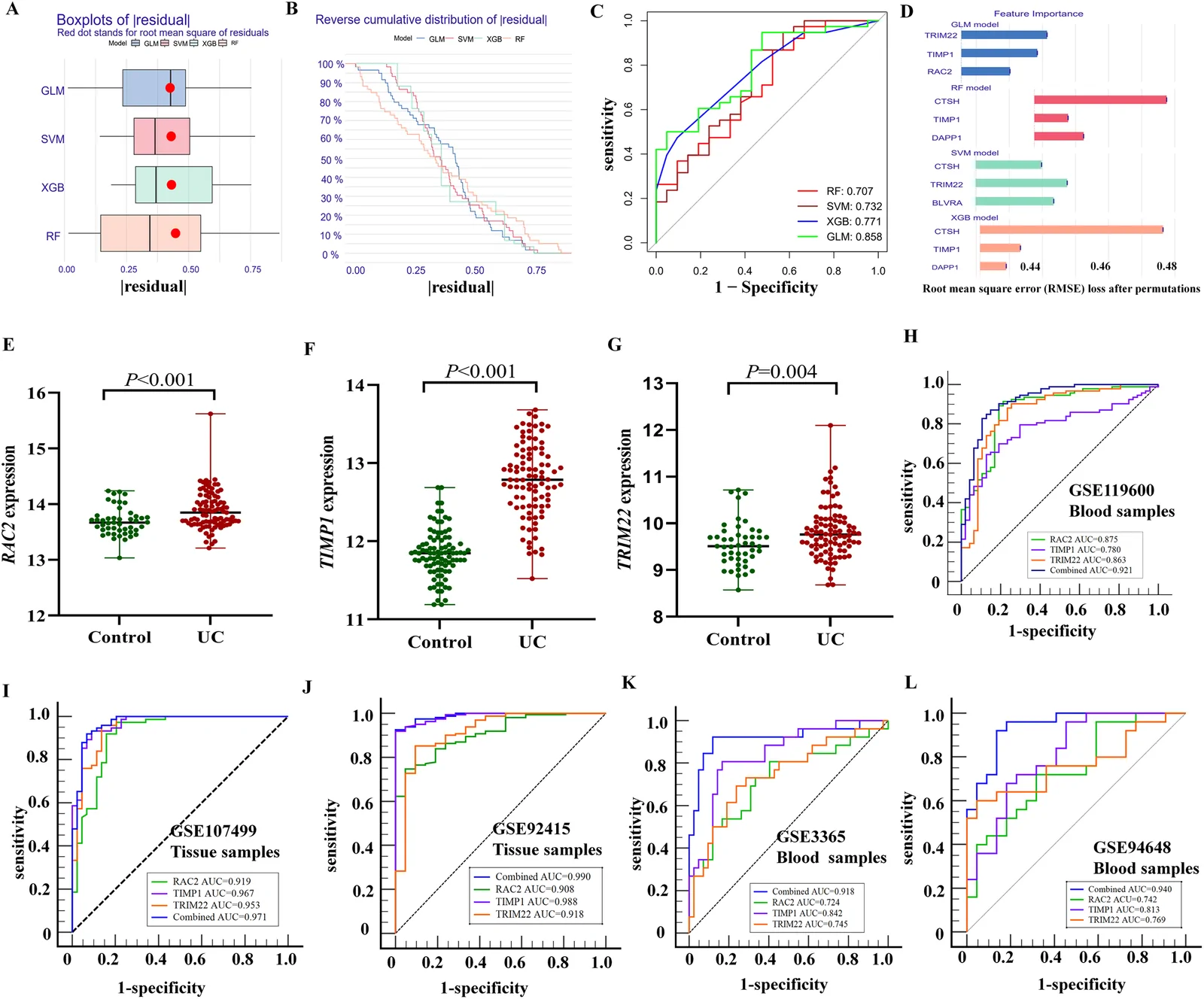
Construction of machine learning model. (A) Boxplots showed the residuals for each machine learning model. (B) Cumulative residual distribution of machine learning model. (C) ROC curves. (D) Important features in GLM, RF, SVM and XGB machine learning models. (E) Validation of Expression of *RAC2* in peripheral blood in GSE119600 dataset. (F) Validation of Expression of *TIMP1* in peripheral blood in GSE119600 dataset. (G) Validation of Expression of *TRIM22* in peripheral blood in GSE119600 dataset. (H) ROC analysis in the GSE119600 dataset. (I) ROC analysis in the GSE107499 dataset. (J) ROC analysis in the GSE92415 dataset. (K) ROC analysis in the GSE3365 dataset. (L) ROC analysis in the GSE94648 dataset.

Four machine learning methods were used to further identify the signature genes: RF, SVM, XGB and the GLM. The residual boxplots showed that the residuals of the four machine learning methods were between 0.25 and 0.50 (Fig. 4A). The cumulative residual distribution plots exhibited consistent results (Fig. 4B). Subsequently, the top three significant feature variables for each model were ranked (Fig. 4D). Based on 5-fold cross validation, the discrimination performance of the four machine learning algorithms was evaluated by the ROC curve (Fig. 4C). The area under the curve (AUC) results of the ROC curve showed that the GLM model had the highest diagnostic value with an AUC of 0.858. Therefore, the GLM machine learning model had the highest efficiency, and the top three key genes identified by GLM model were *TRIM22*, *TIMP1* and *RAC2*.

The expression of these three signature genes was then validated in the GSE119600 dataset (blood samples). The results showed that *RAC2*, *TIMP1*, and *TRIM22* mRNA expression was significantly increased in the peripheral blood of UC patients compared with that of healthy controls (Figs. 4E–4G). The ROC curve analysis showed that the AUC of the combination of the three signature genes for the diagnosis of UC was 0.875 (Fig. 4H). The clinical significance of the three signature genes was further validated in the GSE107499 and GSE92415 datasets (tissue samples), and the AUCs of the combination of the three signature genes for the diagnosis of UC in these datasets were 0.919 and 0.990, respectively (Figs. 4I, 4J). In the blood datasets GSE3365 and GSE94648, the AUCs was 0.918 and 0.940, respectively (Figs. 4K, 4L). ROC analysis of these independent datasets further demonstrated the diagnostic performance of the selected genes and their combination (Table S5). Notably, although *TIMP1* showed a relatively strong differential-expression pattern, its standalone diagnostic performance was not always the strongest across validation datasets, likely because ROC-based classification performance depends not only on group-level expression differences but also on within-group variability, distributional overlap, and dataset-specific heterogeneity.

### Functional enrichment analysis and immune cell analysiss

**Figure 5:**
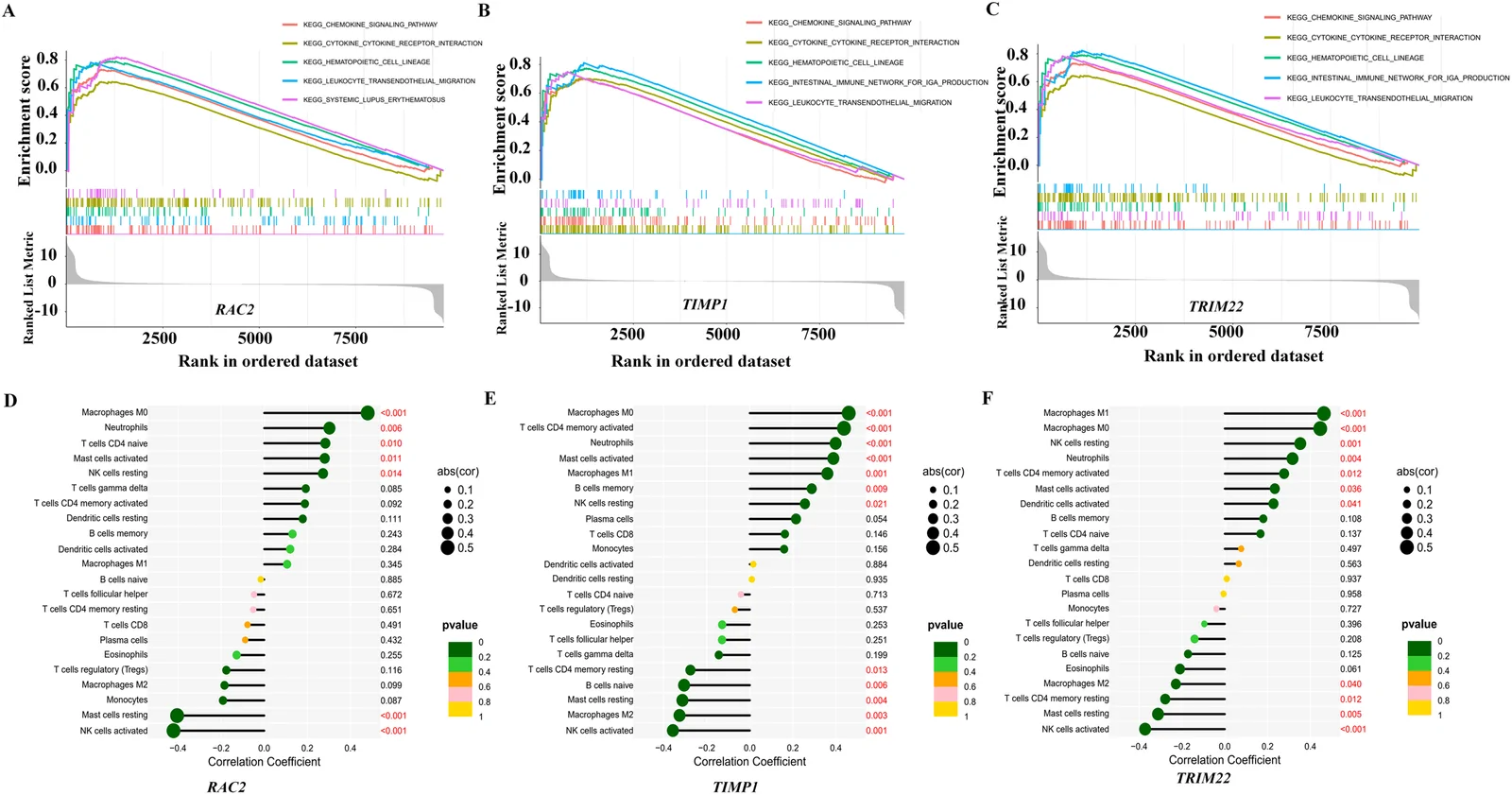
GSEA and immune cell infiltration analysis of key genes. Single gene GSEA analysis for key gene (A) *RAC2*. (B) *TIMP1*. (C) *TRIM22*. Immune cell analysis with key genes. (D) *RAC2*. (E) *TIMP1*. (F) *TRIM22*.

We next investigated the pathway context and immune associations of the three signature genes using GSEA stratified by median expression and immune-cell-fraction analysis (Fig. 5). High expression of RAC2, TIMP1, and TRIM22 was mainly associated with the chemokine signaling pathway, cytokine-receptor interaction, leukocyte transendothelial migration, and intestinal immune network pathways in UC (Figs. 5A–5C), which are associated with UC development. Immune-context analysis showed that RAC2 had significant positive associations with M0 macrophages, neutrophils, naïve CD4 T cells, activated mast cells, and resting NK cells, and negative associations with resting mast cells and activated NK cells (Fig. 5D). For TIMP1, positive associations were found with M0/M1 macrophages, activated CD4 T cells, and neutrophils, and negative associations with resting CD4 T cells, naïve B cells, and M2 macrophages (Fig. 5E). TRIM22 was positively correlated with M0/M1 macrophages, neutrophils, and memory CD4 T cells, and negatively correlated with M2 macrophages, resting CD4 T cells, resting mast cells, and resting NK cells (Fig. 5F). Together, these findings suggest that the three signature genes are closely associated with the UC immune microenvironment.

### Results of single cell analysis

**Figure 6:**
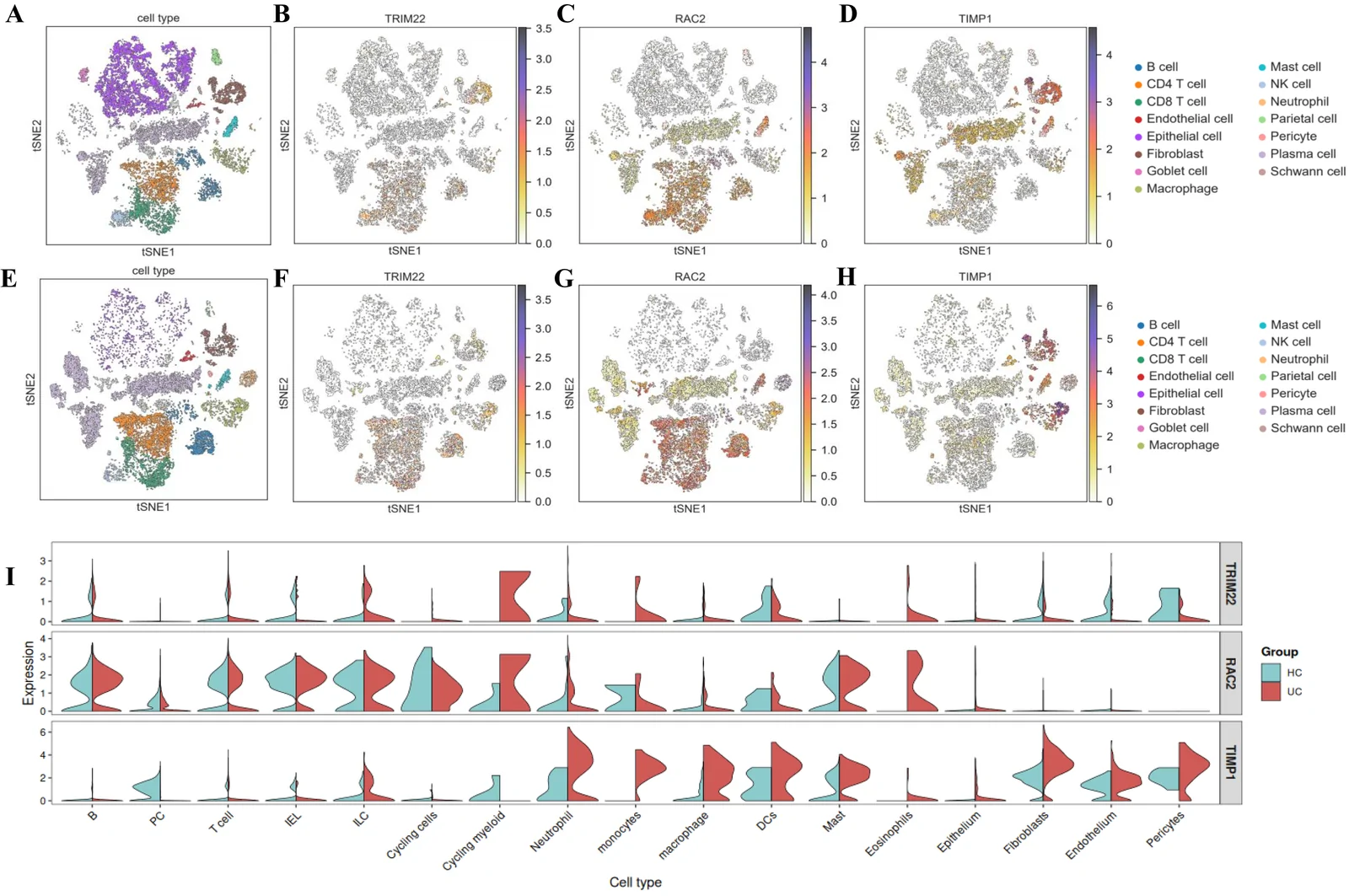
Results of single-cell analysis. (A) t-SNE plot showing annotated cell types in healthy control samples. (B–D) t-SNE plots showing the expression distributions of TRIM22, RAC2, and TIMP1 in healthy control samples, respectively. (E) t-SNE plot showing annotated cell types in UC samples. (F–H) t-SNE plots showing the expression distributions of TRIM22, RAC2, and TIMP1 in UC samples, respectively. (I) Split violin plots comparing TRIM22, RAC2, and TIMP1 expression across major cell types between the healthy control and UC groups.

A single-cell analysis was performed on the GSE214695 dataset, and 15 major cell types were annotated in UC and control samples (Figs. 6A–6E). The cell-type-specific expression patterns of *TRIM22*, *RAC2*, and *TIMP1* were further visualized in control and UC samples (Figs. 6B–6I). Further analysis showed that *TRIM22* was enriched mainly in macrophage-related compartments and was also detectable in lymphocyte populations. *RAC2* showed prominent expression in mast-cell- and lymphocyte-related populations. *TIMP1* was expressed in both macrophage-related and stromal compartments, especially endothelial cells and fibroblasts. These results support the biological plausibility of the bulk immune-correlation analysis while indicating that the PRGs reflect immune-context associations rather than exclusive expression in a single cell lineage.

### Increased expression of hub genes in the intestines of mice with DSS-induced colitis

**Figure 7:**
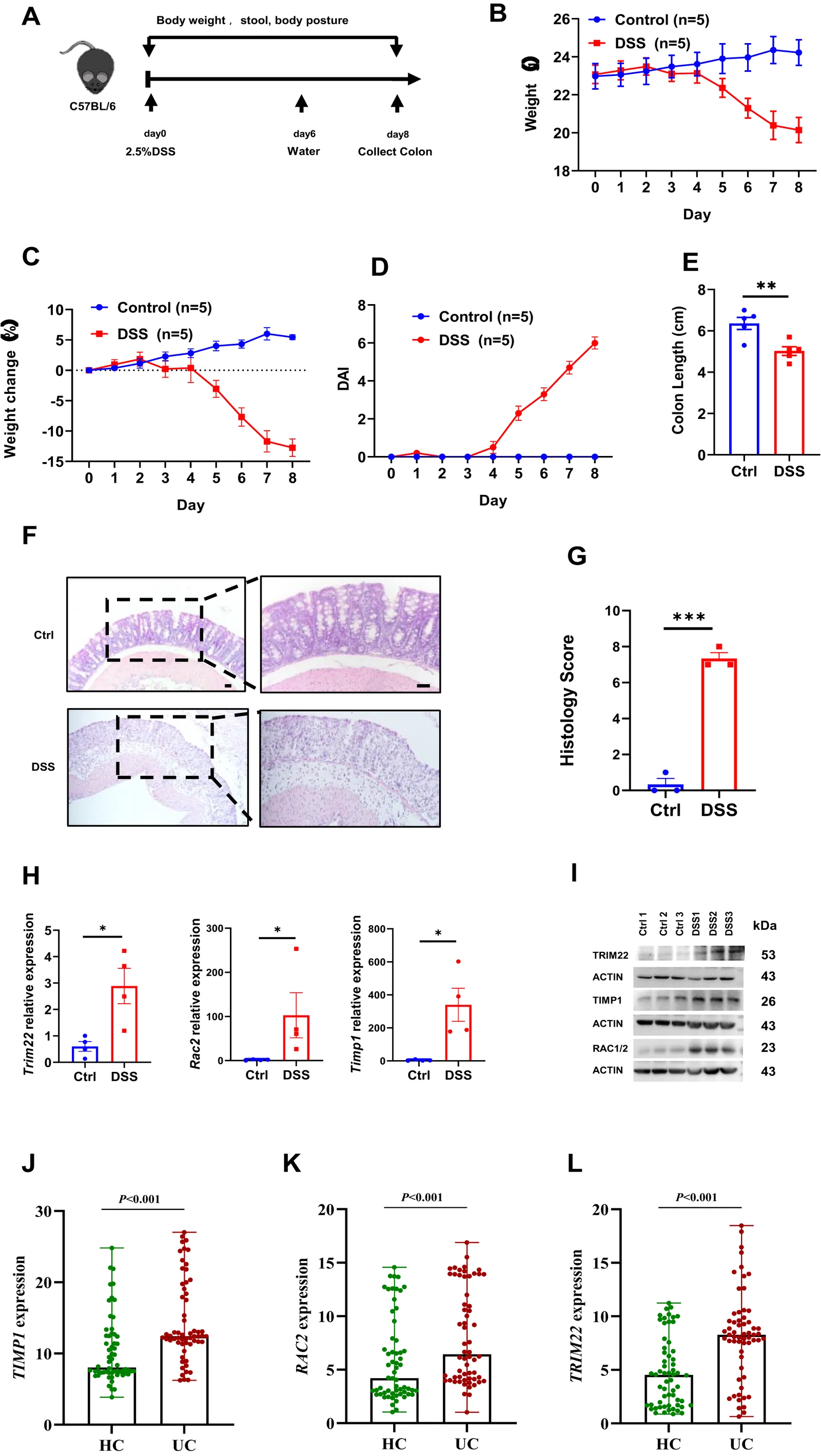
Increased expression of hub genes in UC. (A) Experimental design of DSS-induced colitis. (B) Body weight (C) and body weight change. (D) Disease activity index (DAI) score. (E) Colon lengths. (F) Representative H&E staining (G) of distal colon sections and histology scores. Scale bars, 50 mm. (H) qRT-PCR analysis of the relative mRNA expression of *Trim22*, *Rac2*, and *Timp1* mRNA levels in colonic tissues of control and DSS treated mice on day 8 after DSS treatment. (I) Western blot analysis of the expression of TRIM22, RAC2, and TIMP1 protein in colonic tissues of control and DSS treated mice on day 8 after DSS treatment. (J–L) Expression of mRNA in clinical UC blood PBMC samples and healthy controls (J) *TRIM22*; (K) *RAC2*; (L) *TIMP1*. Groups were compared by two-tailed unpaired t tests. Error bars represent means ± SEM. *P < 0.05, **P < 0.01, ***P < 0.001.

To provide independent *in vivo* validation in an experimental colitis context, we established a DSS-induced colitis mouse model and examined the tissue-level expression of the three hub genes in diseased colons (Figs. 7A, 7B). DSS-treated mice showed significantly lower body weights, higher DAI scores, and shorter colon lengths (Figs. 7C–7E). Furthermore, H&E staining showed disappearance of crypt glands, mucosal damage, inflammatory cell infiltration, and higher histology scores in the colons of DSS-treated mice, whereas mice in the control group exhibited intact colonic mucosa and clear crypts (Figs. 7F, 7G). qRT-PCR and western blot analyses also showed that the mRNA and protein expression of TIMP1, RAC2, and TRIM22 was significantly increased in the colons of DSS-treated mice compared to those of control mice (Figs. 7H, 7I). Collectively, these results suggest that the expression of these three PRGs was significantly increased in the mice with DSS-induced colitis compared with controls.

### Increased expression of hub genes in PBMCs from UC patients

To further validate the expression of the three key genes, peripheral blood samples were used to obtain PBMC samples from 60 UC patients and 60 healthy controls. The clinical data of UC patients and healthy controls were shown in Table 1. There were no significant differences in age, gender, or liver and kidney function biomarkers between the two groups (*P* > 0.05). Compared with controls, UC patients exhibited significantly higher values of peripheral white blood cell counts (*P* = 0.017), neutrophils (*P* = 0.019), monocytes (*P* < 0.001), platelets (*P* = 0.010), CRP (*P* < 0.001) and erythrocyte sedimentation rate (*P* < 0.001) levels. In addition, qRT-PCR analysis showed that the expression of the three signature genes in UC patients was significantly increased in UC compared with the healthy controls (all *P* < 0.05, Figs. 7J–7L).

**Table 1:** Clinical characteristics of the UC patients and healthy controls.

Variables	Total (*n* = 120)	HC (*n* = 60)	UC (*n* = 60)	Statistic	*P*
Age (years)	42.50 (30.75, 56.00)	45.50 (32.00, 56.25)	37.00 (29.00, 50.50)	Z = −1.64	0.102
Gender				χ^2^ = 2.17	0.141
Female	52 (43.33)	22 (36.67)	30 (50.00)		
Male	68 (56.67)	38 (63.33)	30 (50.00)		
WBC (10^9^/L)	6.40 (5.20, 7.85)	6.26 (5.00, 7.29)	7.08 (5.26, 8.47)	Z = −2.39	0.017
LY (10^9^/L)	1.83 (1.54, 2.28)	1.96 (1.62, 2.34)	1.75 (1.41, 2.23)	Z = −1.45	0.147
MO (10^9^/L)	0.44 (0.33, 0.57)	0.37 (0.31, 0.46)	0.51 (0.39, 0.67)	Z = −4.63	<0.001
NE (10^9^/L)	3.79 (2.84, 5.14)	3.54 (2.78, 4.67)	4.22 (3.07, 5.90)	Z = −2.35	0.019
RBC (10^12^/L)	4.56 (4.29, 5.01)	4.66 (4.35, 5.06)	4.52 (4.21, 4.96)	Z = −1.15	0.248
HGB (g/L)	137.50 (123.75, 149.00)	139.00 (130.75, 151.75)	132.00 (116.75, 143.25)	Z = −2.89	0.004
PLT (10^9^/L)	255.00 (207.50, 302.25)	243.50 (203.50, 276.25)	279.50 (224.75, 336.00)	Z = −2.56	0.010
CRP (mg/L)	3.00 (1.13, 8.41)	1.21 (0.49, 2.55)	8.14 (3.51, 15.48)	Z = −7.12	<0.001
ESR (mm/h)	9.50 (4.00, 20.25)	5.00 (3.00, 8.50)	20.50 (10.50, 42.00)	Z = −6.57	<0.001
ALT (U/L)	15.00 (9.30, 23.80)	15.00 (9.23, 24.20)	14.45 (9.90, 23.80)	Z = −0.19	0.846
AST (U/L)	18.65 (15.33, 24.95)	19.05 (15.98, 24.43)	18.15 (15.33, 24.95)	Z = −0.36	0.719
Urea (mmol/L)	4.50 (3.58, 5.43)	4.50 (3.83, 5.45)	4.53 (3.56, 5.28)	Z = −0.22	0.823
Cr (μmol/L)	67.00 (54.88, 74.80)	65.30 (54.47, 74.80)	67.10 (59.90, 74.53)	Z = −0.99	0.324

**Notes:**

WBC, white blood cell; N, neutrophil; L, lymphocyte; M, monocyte; PLT, platelet; ALT, alanine aminotransferase; AST, aspartate aminotransferase; Cr, creatinine.

Z: Mann-Whitney test, χ^2^: Chi-square test.

## Discussion

Platelets have been increasingly implicated in inflammatory diseases such as atherosclerosis, infection, graft rejection, and rheumatoid arthritis (Morrell et al., 2014). In UC, platelet abnormalities are not limited to increased platelet counts; clinical and translational studies have also reported altered platelet indices, enhanced platelet activation markers such as P-selectin/CD62P and CD40L, and increased platelet–monocyte/neutrophil interactions (Chen et al., 2021; Pamuk et al., 2006; Sano et al., 2024). Activated platelets can release inflammatory mediators, including IL-1β, RANTES, and TXA2, and can interact with leukocytes and endothelial cells to promote immune-cell recruitment to inflamed mucosa (Pamuk et al., 2006; Sano et al., 2024; Morrell et al., 2014; Ludwig et al., 2022). In addition, platelets participate in tissue repair, angiogenesis, and mucosal healing, which further supports the biological rationale for introducing a PRG-centered framework into UC research.

In this study, through comprehensive bioinformatics analysis and machine learning algorithms, we successfully identified 3 PRGs (*TRIM22*, *TIMP1*, and *RAC2*) for UC. Although the PRGs cannot be simply regarded as entirely new UC-related genes, the present study provides new insights through their combined value and systematic analysis of immunological characteristics under the framework platelet-related genes. Notably, while *TRIM22* and *TIMP1* have previously been reported to be upregulated in UC, *RAC2* has been much less extensively studied in this disease. Moreover, the clinical significance of the three PRGs still needs to be further explored. Thus, our study extends previous single-gene observations by evaluating these three genes together as a candidate biomarker signature in UC.

*TRIM22* is constitutively highly expressed in monocytes and plays an important role in inflammatory and antiviral responses. Studies have shown that *TRIM22* could participate in the inflammatory response through NF-κB signaling pathway (Ye & Lu, 2022). Moreover, *TRIM22* can promote cell proliferation and inflammation, and inhibition of autophagy through the activation of *PI3K/Akt/mTOR* pathway (Ren et al., 2022). Therefore, the researchers suggested that silencing *TRIM22* gene could be a therapeutic strategy for inflammatory disease (Kang et al., 2021). In the context related to platelets, the platelet-derived growth factor (PDGF), which is mainly synthesized by megakaryocytes and stored in platelets, can be released upon stimulation and participate in inflammatory disease processes. Evidence that TRIM22 can modulate PDGF-related inflammatory responses suggests a possible indirect link between TRIM22 and platelet-derived inflammatory growth-factor signaling (Li & Si, 2025). In this study, we systematically examined the relationship between the PRGs and the immune features of UC. *TRIM22* was positively correlated with M0/M1 macrophages, neutrophils, and memory CD4 T cells, and negatively correlation with M2 macrophages. Therefore, TRIM22 may be more closely related to immune signaling and macrophage/neutrophil-associated inflammation (Hossain, 2018).

As for the *TIMP1*, which belongs to the tissue inhibitor of metalloproteinases (MMPs) family, is a multifunctional gene (Schoeps et al., 2021). Highly expressed *TIMP1* could promote cell proliferation and invasion through Akt pathway (Ma et al., 2022; Wang, Wang & Chen, 2023). Like *TRIM22*, *TIMP1* showed positive association with M0/M1 macrophages, activated CD4 T cells, and neutrophils. The single cell analysis further revealed that *TIMP1* was expressed in both macrophage-related and stromal compartments, especially endothelial cells and fibroblasts. Therefore, *TIMP1* may reflect extracellular matrix remodeling and fibrosis-prone tissue response (Huang et al., 2019; Jakubowska et al., 2016; Czajkowska et al., 2022; Pan et al., 2023; Sato et al., 2021). In the context related to platelets, platelets can store and release matrix metalloproteinases and tissue inhibitors of metalloproteinases, including TIMP-1, and platelet-derived MMPs/TIMPs can regulate platelet function under physiological and pathological conditions (Gresele et al., 2017). Activated platelet-derived microparticles have also been reported to carry angiogenic proteins, including VEGF, angiopoietin-1, and TIMP-1, suggesting a potential link among platelet activation, angiogenic signaling, and tissue remodeling (Gaetani et al., 2018). In addition, TIMP1 mRNA in tumor-educated platelets has been reported as a potential diagnostic biomarker in colorectal cancer, further supporting the relevance of *TIMP1* to platelet-associated molecular signatures (Yang et al., 2019).

The *RAC2* gene (Ras-associated C3 botulinum toxin substrate 2) encodes a small GTPase protein that plays an important regulatory role in the cell, including gene expression, cell proliferation, cell adhesion, and cytoskeleton formation (Liu et al., 2023). Studies had shown that *RAC2* was expressed exclusively in blood-derived cells, and participate in hematopoietic cell formation (Filippi et al., 2004). However, recent report has suggested that *RAC2* gene mutations may lead to the dysfunction of the immune system, leading to some immune-related diseases (Lougaris et al., 2020). *RAC2* played a critical role in the process of granulocyte degranulation and had been involved in neutrophil-mediated damage (Dooley et al., 2009; Ilarraza et al., 2023). Studies have suggested RAC2 may reflect immune-cell migration and cytoskeletal signaling programs (Li et al., 2023; Seinen et al., 2016; Wildenberg et al., 2017; Joshi et al., 2017). Previous evidence has shown that Rac2 GTPase plays an important role in platelet adhesion to fibrinogen and in the maintenance and perpetuation of platelet aggregation, suggesting a connection between RAC2 and platelet functional programs (Akbar et al., 2004). In our analysis, *RAC2* had significant positive associations with inflammatory cells such as M0 macrophages, neutrophils, this suggested that the interaction between *RAC2* played an important immunomodulatory role in diseases.

Together, our observations support the view that platelet-related molecular features are linked not only to hemostatic processes but also to the inflammatory and immune microenvironment of UC. Notably, *TRIM22*, *TIMP1*, and *RAC2* should be understood as platelet-associated inflammatory or immunothrombotic candidate genes that capture distinct but complementary components of the UC inflammatory microenvironment, rather than as platelet-specific transcripts (Akbar et al., 2004). In our analysis, we validated the expression patterns of these three genes in PBMC samples from UC patients and in DSS-induced colitis tissues, further supporting their potential utility as a combined biomarker signature in UC.

Since the above immune-fraction and PRG-correlation analyses were based on bulk transcriptomic data and could not directly determine the cellular origin of these associations, single-cell analysis was further used to define the cell-type-specific expression landscape of the three hub genes and to cross-check the biological plausibility of the bulk immune findings. Rather than implying that these genes are restricted to a single immune lineage, the single-cell data showed that *TRIM22* was enriched in macrophage-related compartments, *TIMP1* was distributed across both macrophage-related and stromal compartments, and *RAC2* was enriched in mast-cell- and lymphocyte-related populations. These results support biologically plausible immune-context associations while also indicating that the bulk CIBERSORT correlations should not be interpreted as evidence of exclusive cellular origin.

Although our study highlights the diagnostic and biological relevance of a PRG-centered model in UC, several limitations should be noted. First, although six GEO cohorts were included, the sample size and inter-dataset heterogeneity remained limited, and the AUC values across independent validation datasets varied, likely in part because of differences in sample size, platform background, and cohort characteristics. Second, although we used PBMC samples from UC patients and a DSS-induced colitis model for independent biological validation, the clinical significance of the signature genes still requires evaluation in larger and more deeply phenotyped UC cohorts. Third, the machine-learning analysis was designed as an exploratory feature-prioritization step based on a fixed train-test split strategy. Although the stability of feature selection was validated using single train-test split on the GSE11223 dataset and on independent external datasets; more rigorous validation—such as nested cross-validation—will be employed in future work to robustly assess methodological stability. Fourth, although we identified three key genes through machine-learning analysis, their detailed molecular mechanisms were not explored experimentally in this study. Additional downstream analyses, such as competitive regulatory network construction or drug-sensitivity prediction, would be promising next steps to extend these observations. Future work should therefore evaluate the clinical relevance, upstream regulatory mechanisms, and therapeutic implications of *TRIM22*, *TIMP1*, and *RAC2* in UC.

## Conclusion

In conclusion, our study established a platelet-related gene-centered framework in UC and identified a candidate three-gene signature consisting of *TRIM22*, *TIMP1*, and *RAC2*. Rather than proposing these genes as entirely unprecedented UC molecules, our work provides new biological and clinical insight by integrating platelet-related prioritization, immune-context interpretation, single-cell localization, and multi-context validation across tissue, blood, PBMC, and DSS-colitis settings.

## Supplemental Information

10.7717/peerj.21655/supp-1 Supplemental Information 1 Computer code and descriptive legends.

10.7717/peerj.21655/supp-2 Supplemental Information 2 Raw data for Figure 7.

10.7717/peerj.21655/supp-3 Supplemental Information 3 Code about DEGs, CIBERSORT, MLROC, SCSEQ, GSEA. Code_DEG_analysis.R：使用 R 语言进行血小板相关基因差异表达分析并生成 DEG 相关图的代码.Code_CIBERSORT_analysis.R：使用 R 语言进行基于 CIBERSORT 的免疫细胞比例估计和免疫浸润可视化的代码.Code_ML_modeling.R：使用 R 语言构建机器学习模型的代码.Code_SCSEQ_analysis.R：使用 R 语言进行单细胞 RNA 测序分析的代码. Code_GSEA_analysis.R：使用 R 语言进行GSEA分析的代码.

10.7717/peerj.21655/supp-4 Supplemental Information 4 Original image for WB.

10.7717/peerj.21655/supp-5 Supplemental Information 5 Details of the sample information of the datasets.

10.7717/peerj.21655/supp-6 Supplemental Information 6 485 platelet-related genes.

10.7717/peerj.21655/supp-7 Supplemental Information 7 Sequences of primers used for qRT-PCR.

10.7717/peerj.21655/supp-8 Supplemental Information 8 37 differential PRGs.

10.7717/peerj.21655/supp-9 Supplemental Information 9 Independent validation ROC summary.

10.7717/peerj.21655/supp-10 Supplemental Information 10 Flow chart of this study.

10.7717/peerj.21655/supp-11 Supplemental Information 11 Immune cell infiltration analysis between UC and control group. (A) Boxplot showed the differences of 22 infiltrated immune cells between UC and control group. (B) Relative abundances of 22 infiltrated immune cells between UC and controls. (C) Correlation analysis between PRGs and 22 immune cells. (D) Correlation analysis showed the positive association between *TRIM22* and M0 macrophages. (E) Positive association of *TRIM22* and M1 macrophages. (F) Negative association of *TRIM22* and M2 macrophages. (G) Negative association between *TRIM22* and resting CD4 T memory cell.

10.7717/peerj.21655/supp-12 Supplemental Information 12 MIQE checklist.

10.7717/peerj.21655/supp-13 Supplemental Information 13 ARRIVE checklist.

10.7717/peerj.21655/supp-14 Supplemental Information 14 Processed result files from DEG, CIBERSORT, WGCNA, machine-learning, and ROC analyses. Processed input and output result files organized into DEG, CIBERSORT, WGCNA, machine-learning, and ROC analysis folders. It includes differential-expression results, immune-cell fraction estimates, WGCNA module gene lists from disease-related and cluster-related co-expression analyses, model variable-importance outputs, and ROC diagnostic-performance results. This file contains processed data/results only and does not contain executable code.

## Additional Information and Declarations

### Data Availability

The following information was supplied regarding data availability:

The code and raw measurements are available in the Supplemental Files.
