# Supplementary material for: Identification and validation of novel platelet-related genes in ulcerative colitis using bioinformatics and machine learning strategies
Source: PeerJ. 2026 Aug 25;14:e21655. doi: 10.7717/peerj.21655 (PMC13525761; doi:10.7717/peerj.21655)
Supplement: Supplementary file 1 [file peerj-14-21655-s010.pdf]

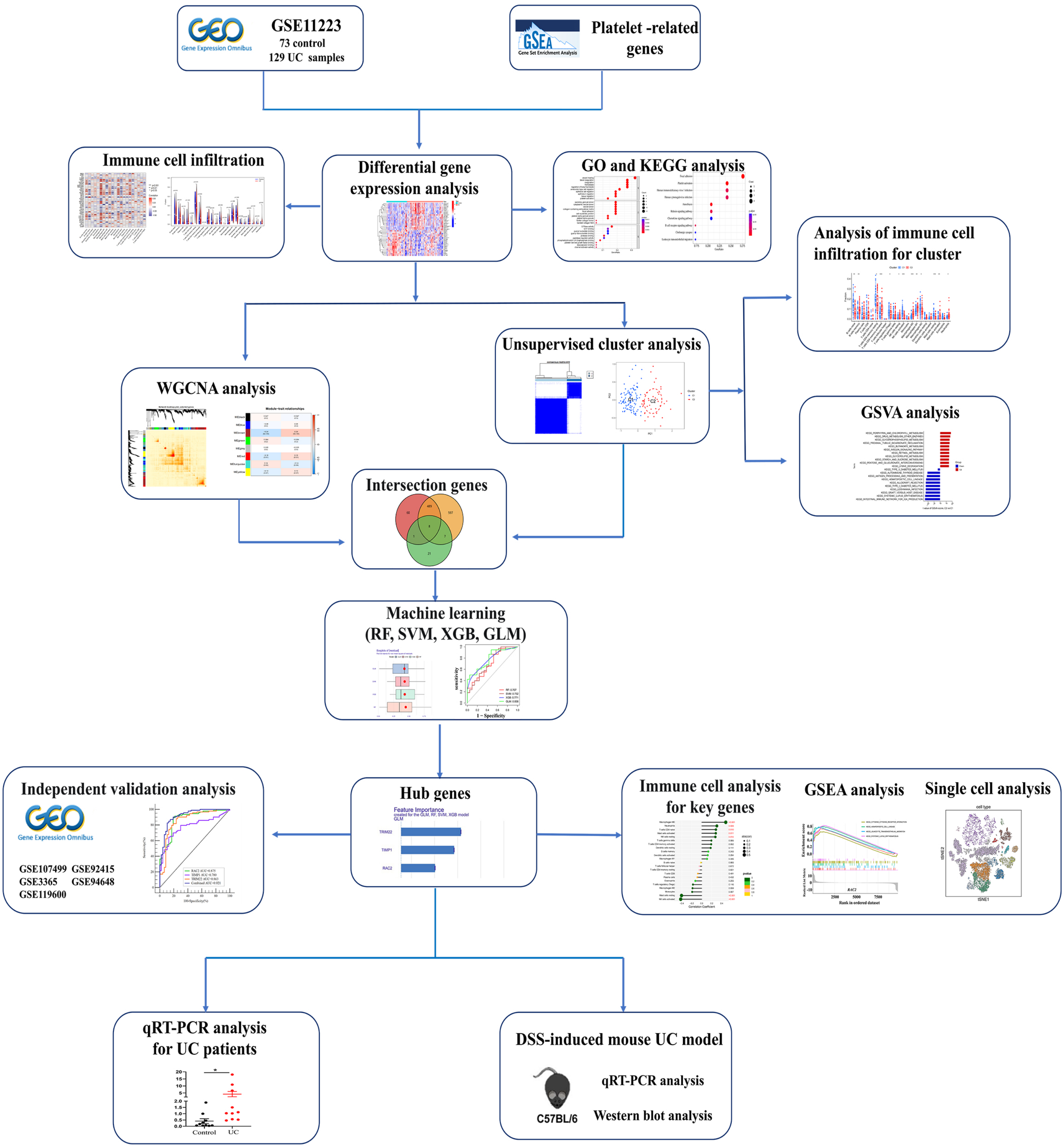
GSE11223
73 control
129 UC samples
Platelet -related genes
Immune cell infiltration
Differential gene expression analysis
GO and KEGG analysis
Analysis of immune cell infiltration for cluster
WGCNA analysis
Unsupervised cluster analysis
GSVA analysis
Intersection genes
Machine learning
(RF, SVM, XGB, GLM)
Independent validation analysis
GSE107499 GSE92415
GSE3365 GSE94648
GSE119600
Hub genes
Feature Importance
TRIM22
TIMP1
RAC2
Immune cell analysis for key genes
GSEA analysis
Single cell analysis
qRT-PCR analysis for UC patients
Control
UC
DSS-induced mouse UC model
qRT-PCR analysis
Western blot analysis
C57BL/6
