# Supplementary material for: Identification and validation of novel platelet-related genes in ulcerative colitis using bioinformatics and machine learning strategies
Source: PeerJ. 2026 Aug 25;14:e21655. doi: 10.7717/peerj.21655 (PMC13525761; doi:10.7717/peerj.21655)
Supplement: Supplementary file 2 [file peerj-14-21655-s011.pdf]

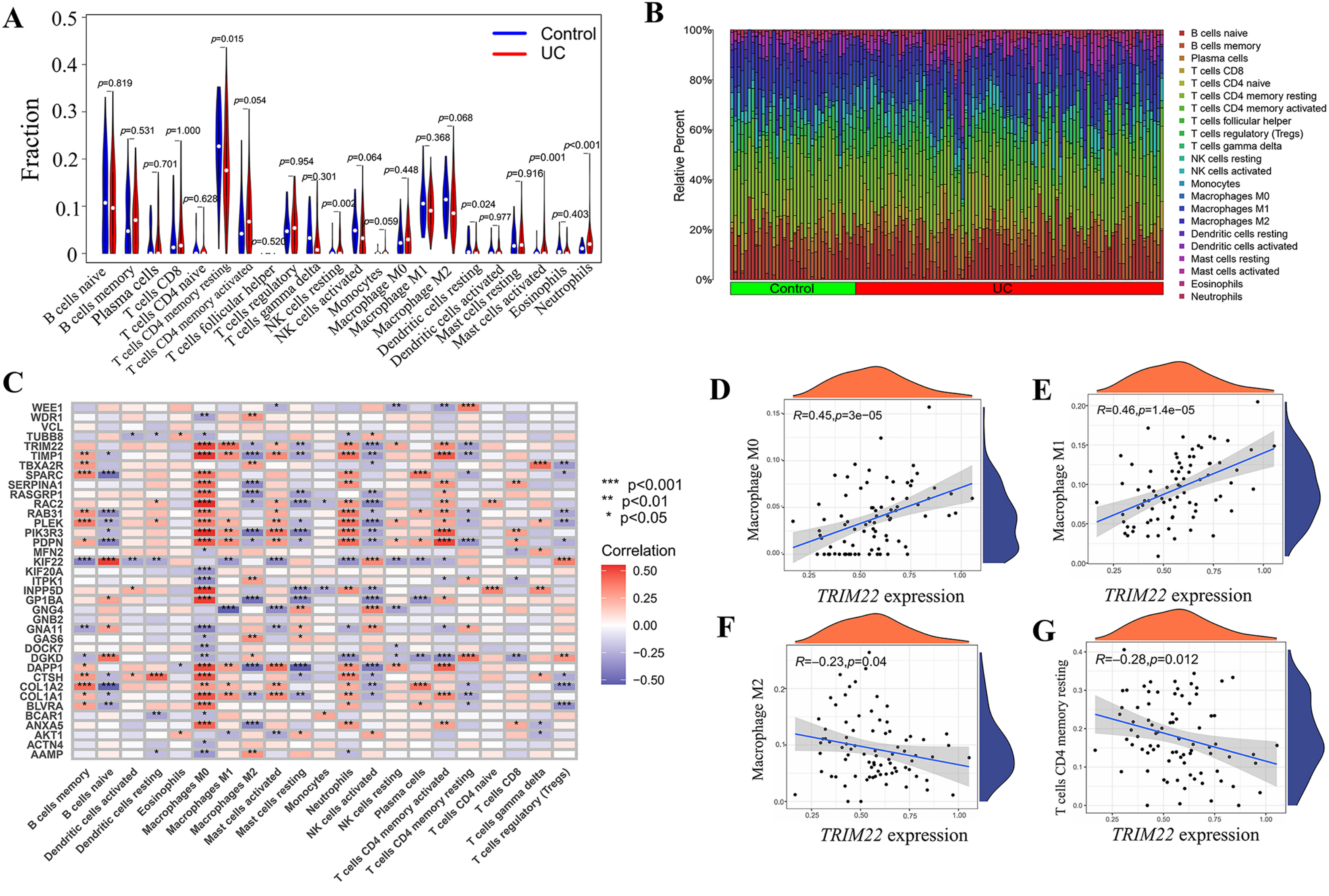

A
Fraction
Control
UC
p=0.819
p=0.531
p=0.701
p=1.000
p=0.628
p=0.015
p=0.054
p=0.520
p=0.954
p=0.301
p=0.002
p=0.064
p=0.059
p=0.448
p=0.368
p=0.068
p=0.024
p=0.977
p=0.916
p=0.001
p=0.403
p<0.001
B cells naive
B cells memory
Plasma cells
T cells CD8
T cells CD4 naive
T cells CD4 memory resting
T cells CD4 memory activated
T cells follicular helper
T cells regulatory
T cells gamma delta
NK cells resting
NK cells activated
Monocytes
Macrophage M0
Macrophage M1
Macrophage M2
Dendritic cells resting
Dendritic cells activated
Mast cells resting
Mast cells activated
Eosinophils
Neutrophils
B
Relative Percent
100%
80%
60%
40%
20%
0%
Control
UC
B cells naive
B cells memory
Plasma cells
T cells CD8
T cells CD4 naive
T cells CD4 memory resting
T cells CD4 memory activated
T cells follicular helper
T cells regulatory (Tregs)
T cells gamma delta
NK cells resting
NK cells activated
Monocytes
Macrophages M0
Macrophages M1
Macrophages M2
Dendritic cells resting
Dendritic cells activated
Mast cells resting
Mast cells activated
Eosinophils
Neutrophils
C
WEE1
WDR1
VCL
TUBB8
TRIM22
TIMP1
TBXA2R
SPARC
SERPINA1
RASGRP1
RAC2
RAB31
PLEK
PIK3R3
PDPN
MFN2
KIF22
KIF20A
ITPK1
INPP5D
GP1BA
GNG4
GNB2
GNA11
GAS6
DOCK7
DGKD
DAPP1
CTSH
COL1A2
COL1A1
BLVRA
BCAR1
ANXA5
AKT1
ACTN4
AAMP
B cells memory
B cells naive
Dendritic cells activated
Dendritic cells resting
Eosinophils
Macrophages M0
Macrophages M1
Macrophages M2
Mast cells activated
Mast cells resting
Monocytes
Neutrophils
NK cells activated
NK cells resting
Plasma cells
T cells CD4 memory activated
T cells CD4 memory resting
T cells CD4 naive
T cells CD8
T cells gamma delta
T cells regulatory (Tregs)
*** p<0.001
** p<0.01
* p<0.05
Correlation
0.50
0.25
0.00
−0.25
−0.50
D
R=0.45,p=3e−05
Macrophage M0
TRIM22 expression
E
R=0.46,p=1.4e−05
Macrophage M1
TRIM22 expression
F
R=−0.23,p=0.04
Macrophage M2
TRIM22 expression
G
R=−0.28,p=0.012
T cells CD4 memory resting
TRIM22 expression
