# Supplementary material for: Identification and validation of novel platelet-related genes in ulcerative colitis using bioinformatics and machine learning strategies
Source: PeerJ. 2026 Aug 25;14:e21655. doi: 10.7717/peerj.21655 (PMC13525761; doi:10.7717/peerj.21655)
Supplement: Supplementary file 4 [file peerj-14-21655-s001.docx]

1. Normalize.R: Gene Expression Data Normalization and Preprocessing

This script performs normalization and preprocessing of raw gene expression data. Its primary functions include: reading the raw expression matrix (geneMatrix.txt) and sample grouping information (s1.txt, s2.txt); automatically determining the need for log2 transformation based on data distribution characteristics; and applying quantile normalization using the normalizeBetweenArrays() function from the limma package to eliminate technical batch effects. The output file normalize.txt provides standardized expression data for downstream analyses.

library(limma)

expFile="geneMatrix.txt"

conFile="s1.txt"

treatFile="s2.txt"

rt=read.table(expFile, header=T, sep="\t", check.names=F)

rt=as.matrix(rt)

rownames(rt)=rt[,1]

exp=rt[,2:ncol(rt)]

dimnames=list(rownames(exp),colnames(exp))

data=matrix(as.numeric(as.matrix(exp)),nrow=nrow(exp),dimnames=dimnames)

data=avereps(data)

s1=read.table(conFile, header=F, sep="\t", check.names=F)

sampleName1=as.vector(s1[,1])

conData=data[,sampleName1]

s2=read.table(treatFile, header=F, sep="\t", check.names=F)

sampleName2=as.vector(s2[,1])

treatData=data[,sampleName2]

rt=cbind(conData, treatData)

qx=as.numeric(quantile(rt, c(0, 0.25, 0.5, 0.75, 0.99, 1.0), na.rm=T))

LogC=( (qx[5]>100) || ( (qx[6]-qx[1])>50 && qx[2]>0) )

if(LogC){

rt[rt<0]=0

rt=log2(rt+1)}

data=normalizeBetweenArrays(rt)

conNum=ncol(conData)

treatNum=ncol(treatData)

Type=c(rep("Control",conNum),rep("Treat",treatNum))

outData=rbind(id=paste0(colnames(data),"_",Type),data)

write.table(outData, file="normalize.txt", sep="\t", quote=F, col.names=F)

2. diff.R: Differential Expression Gene Screening and Visualization

This script conducts differential expression analysis on normalized expression data. Using the non-parametric Wilcoxon rank-sum test, it identifies genes with significant differential expression between groups (p < 0.05) and incorporates multiple visualization approaches: a heatmap displays the overall expression patterns of differentially expressed genes (with row-wise standardization and clustering), while a multi-gene side-by-side boxplot illustrates the expression distribution and statistical significance of each gene across two groups. The final outputs include an expression matrix of significantly differentially expressed genes (diffGeneExp.txt) and high-quality visualization plots.

library(limma)

library(pheatmap)

library(reshape2)

library(ggpubr)

expFile="crgGeneExp.txt"

rt=read.table(expFile, header=T, sep="\t", check.names=F)

rt=as.matrix(rt)

rownames(rt)=rt[,1]

exp=rt[,2:ncol(rt)]

dimnames=list(rownames(exp),colnames(exp))

data=matrix(as.numeric(as.matrix(exp)),nrow=nrow(exp),dimnames=dimnames)

data=avereps(data)

exp=data

Type=gsub("(.*)\\_(.*)", "\\2", colnames(data))

sigVec=c()

sigGeneVec=c()

for(i in row.names(data)){

test=wilcox.test(data[i,] ~ Type)

pvalue=test$p.value

Sig=ifelse(pvalue<0.001,"***",ifelse(pvalue<0.01,"**",ifelse(pvalue<0.05,"*","")))

if(pvalue<0.05){

sigVec=c(sigVec, paste0(i, Sig))

sigGeneVec=c(sigGeneVec, i)}

}

data=data[sigGeneVec,]

outTab=rbind(ID=colnames(data), data)

write.table(outTab, file="diffGeneExp.txt", sep="\t", quote=F, col.names=F)

row.names(data)=sigVec

names(Type)=colnames(data)

Type=as.data.frame(Type)

pdf(file="heatmap.pdf", width=7, height=4.5)

pheatmap(data,

annotation=Type,

color = colorRampPalette(c(rep("blue",2), "white", rep("red",2)))(100),

cluster_cols =F,

cluster_rows =T,

scale="row",

show_colnames=F,

show_rownames=T,

fontsize=7,

fontsize_row=7,

fontsize_col=7)

dev.off()

exp=as.data.frame(t(exp))

exp=cbind(exp, Type=Type)

data=melt(exp, id.vars=c("Type"))

colnames(data)=c("Type", "Gene", "Expression")

p=ggboxplot(data, x="Gene", y="Expression", color = "Type",

xlab="",

ylab="Gene expression",

legend.title="Type",

palette = c("blue", "red"),

add="point",

width=0.8)

p=p+rotate_x_text(60)

p1=p+stat_compare_means(aes(group=Type),

method="wilcox.test",

symnum.args=list(cutpoints = c(0, 0.001, 0.01, 0.05, 1), symbols = c("***", "**", "*", " ")),

label = "p.signif")

pdf(file="boxplot.pdf", width=7, height=5)

print(p1)

dev.off()

3. WGCNA.R： Weighted Gene Co-expression Network Analysis (WGCNA)

This script performs system-level co-expression network construction and module analysis. It begins by screening highly variable genes, selects an appropriate soft threshold to construct a scale-free gene co-expression network, and employs hierarchical clustering based on topological overlap measure to identify gene modules with coordinated expression patterns. Further analyses examine the association between module eigengenes and sample phenotypes (e.g., disease status), compute gene significance (correlation with phenotype) and module membership (correlation with module eigengenes), and ultimately identify hub genes. The script outputs gene lists for each module and comprehensive network analysis results.

library(limma)

library(gplots)

library(WGCNA)

expFile="normalize.txt"

rt=read.table(expFile, header=T, sep="\t", check.names=F)

rt=as.matrix(rt)

rownames(rt)=rt[,1]

exp=rt[,2:ncol(rt)]

dimnames=list(rownames(exp),colnames(exp))

data=matrix(as.numeric(as.matrix(exp)),nrow=nrow(exp),dimnames=dimnames)

data=avereps(data)

selectGenes=names(tail(sort(apply(data,1,sd)), n=round(nrow(data)*0.25)))

data=data[selectGenes,]

Type=gsub("(.*)\\_(.*)", "\\2", colnames(data))

conCount=length(Type[Type=="Control"])

treatCount=length(Type[Type=="Treat"])

datExpr0=t(data)

gsg = goodSamplesGenes(datExpr0, verbose = 3)

if (!gsg$allOK){

\# Optionally, print the gene and sample names that were removed:

if (sum(!gsg$goodGenes)>0)

printFlush(paste("Removing genes:", paste(names(datExpr0)[!gsg$goodGenes], collapse = ", ")))

if (sum(!gsg$goodSamples)>0)

printFlush(paste("Removing samples:", paste(rownames(datExpr0)[!gsg$goodSamples], collapse = ", ")))

\# Remove the offending genes and samples from the data:

datExpr0 = datExpr0[gsg$goodSamples, gsg$goodGenes]

}

sampleTree = hclust(dist(datExpr0), method = "average")

pdf(file = "01.sample_cluster.pdf", width = 12, height = 9)

par(cex = 0.6)

par(mar = c(0,4,2,0))

plot(sampleTree, main = "Sample clustering to detect outliers", sub="", xlab="", cex.lab = 1.5, cex.axis = 1.5, cex.main = 2)

abline(h = 20000, col="red")

dev.off()

clust=cutreeStatic(sampleTree, cutHeight=20000, minSize=10)

table(clust)

keepSamples=(clust==1)

datExpr0=datExpr0[keepSamples,]

traitData=data.frame(Con=c(rep(1,conCount),rep(0,treatCount)),

Treat=c(rep(0,conCount),rep(1,treatCount)))

row.names(traitData)=colnames(data)

fpkmSamples=rownames(datExpr0)

traitSamples=rownames(traitData)

sameSample=intersect(fpkmSamples,traitSamples)

datExpr0=datExpr0[sameSample,]

datTraits=traitData[sameSample,]

sampleTree2 = hclust(dist(datExpr0), method="average")

traitColors = numbers2colors(datTraits, signed = FALSE)

pdf(file="02.sample_heatmap.pdf", width=12, height=12)

plotDendroAndColors(sampleTree2, traitColors,

groupLabels = names(datTraits),

main = "Sample dendrogram and trait heatmap")

dev.off()

enableWGCNAThreads()

powers = c(1:20)

sft = pickSoftThreshold(datExpr0, powerVector = powers, verbose = 5)

pdf(file="03.scale_independence.pdf",width=9,height=5)

par(mfrow = c(1,2))

cex1 = 0.9

plot(sft$fitIndices[,1], -sign(sft$fitIndices[,3])*sft$fitIndices[,2],

xlab="Soft Threshold (power)",ylab="Scale Free Topology Model Fit,signed R^2",type="n",

main = paste("Scale independence"));

text(sft$fitIndices[,1], -sign(sft$fitIndices[,3])*sft$fitIndices[,2],

labels=powers,cex=cex1,col="red");

abline(h=0.90,col="red")

plot(sft$fitIndices[,1], sft$fitIndices[,5],

xlab="Soft Threshold (power)",ylab="Mean Connectivity", type="n",

main = paste("Mean connectivity"))

text(sft$fitIndices[,1], sft$fitIndices[,5], labels=powers, cex=cex1,col="red")

dev.off()

softPower =sft$powerEstimate

adjacency = adjacency(datExpr0, power = softPower)

softPower

TOM = TOMsimilarity(adjacency)

dissTOM = 1-TOM

geneTree = hclust(as.dist(dissTOM), method = "average");

pdf(file="04.gene_clustering.pdf",width=12,height=9)

plot(geneTree, xlab="", sub="", main = "Gene clustering on TOM-based dissimilarity",

labels = FALSE, hang = 0.04)

dev.off()

minModuleSize = 100

dynamicMods = cutreeDynamic(dendro = geneTree, distM = dissTOM,

deepSplit = 2, pamRespectsDendro = FALSE,

minClusterSize = minModuleSize);

table(dynamicMods)

dynamicColors = labels2colors(dynamicMods)

table(dynamicColors)

pdf(file="05.Dynamic_Tree.pdf",width=8,height=6)

plotDendroAndColors(geneTree, dynamicColors, "Dynamic Tree Cut",

dendroLabels = FALSE, hang = 0.03,

addGuide = TRUE, guideHang = 0.05,

main = "Gene dendrogram and module colors")

dev.off()

MEList = moduleEigengenes(datExpr0, colors = dynamicColors)

MEs = MEList$eigengenes

MEDiss = 1-cor(MEs);

METree = hclust(as.dist(MEDiss), method = "average")

pdf(file="06.Clustering_module.pdf",width=7,height=6)

plot(METree, main = "Clustering of module eigengenes",

xlab = "", sub = "")

dev.off()

moduleColors=dynamicColors

nGenes = ncol(datExpr0)

nSamples = nrow(datExpr0)

select = sample(nGenes, size=1000)

selectTOM = dissTOM[select, select];

selectTree = hclust(as.dist(selectTOM), method="average")

selectColors = moduleColors[select]

#sizeGrWindow(9,9)

plotDiss=selectTOM^softPower

diag(plotDiss)=NA

myheatcol = colorpanel(250, "red", "orange", "lemonchiffon")

pdf(file="07.TOMplot.pdf", width=7, height=7)

TOMplot(plotDiss, selectTree, selectColors, main = "Network heatmap plot, selected genes", col=myheatcol)

dev.off()

moduleTraitCor = cor(MEs, datTraits, use = "p")

moduleTraitPvalue = corPvalueStudent(moduleTraitCor, nSamples)

pdf(file="08.Module_trait.pdf", width=6.5, height=5.5)

textMatrix = paste(signif(moduleTraitCor, 2), "\n(",

signif(moduleTraitPvalue, 1), ")", sep = "")

dim(textMatrix) = dim(moduleTraitCor)

par(mar = c(3.5, 8, 3, 3))

labeledHeatmap(Matrix = moduleTraitCor,

xLabels = names(datTraits),

yLabels = names(MEs),

ySymbols = names(MEs),

colorLabels = FALSE,

colors = blueWhiteRed(50),

textMatrix = textMatrix,

setStdMargins = FALSE,

cex.text = 0.7,

zlim = c(-1,1),

main = paste("Module-trait relationships"))

dev.off()

modNames = substring(names(MEs), 3)

geneModuleMembership = as.data.frame(cor(datExpr0, MEs, use = "p"))

MMPvalue = as.data.frame(corPvalueStudent(as.matrix(geneModuleMembership), nSamples))

names(geneModuleMembership) = paste("MM", modNames, sep="")

names(MMPvalue) = paste("p.MM", modNames, sep="")

traitNames=names(datTraits)

geneTraitSignificance = as.data.frame(cor(datExpr0, datTraits, use = "p"))

GSPvalue = as.data.frame(corPvalueStudent(as.matrix(geneTraitSignificance), nSamples))

names(geneTraitSignificance) = paste("GS.", traitNames, sep="")

names(GSPvalue) = paste("p.GS.", traitNames, sep="")

trait="Treat"

traitColumn=match(trait,traitNames)

for (module in modNames){

column = match(module, modNames)

moduleGenes = moduleColors==module

if (nrow(geneModuleMembership[moduleGenes,]) > 1){

outPdf=paste("09.", trait, "_", module,".pdf",sep="")

pdf(file=outPdf,width=7,height=7)

par(mfrow = c(1,1))

verboseScatterplot(abs(geneModuleMembership[moduleGenes, column]),

abs(geneTraitSignificance[moduleGenes, traitColumn]),

xlab = paste("Module Membership in", module, "module"),

ylab = paste("Gene significance for ",trait),

main = paste("Module membership vs. gene significance\n"),

cex.main = 1.2, cex.lab = 1.2, cex.axis = 1.2, col = module)

abline(v=0.8,h=0.5,col="red")

dev.off()

}

}

probes = colnames(datExpr0)

geneInfo0 = data.frame(probes= probes,

moduleColor = moduleColors)

for (Tra in 1:ncol(geneTraitSignificance))

{

oldNames = names(geneInfo0)

geneInfo0 = data.frame(geneInfo0, geneTraitSignificance[,Tra],

GSPvalue[, Tra])

names(geneInfo0) = c(oldNames,names(geneTraitSignificance)[Tra],

names(GSPvalue)[Tra])

}

for (mod in 1:ncol(geneModuleMembership))

{

oldNames = names(geneInfo0)

geneInfo0 = data.frame(geneInfo0, geneModuleMembership[,mod],

MMPvalue[, mod])

names(geneInfo0) = c(oldNames,names(geneModuleMembership)[mod],

names(MMPvalue)[mod])

}

geneOrder =order(geneInfo0$moduleColor)

geneInfo = geneInfo0[geneOrder, ]

write.table(geneInfo, file = "GS_MM.xls",sep="\t",row.names=F)

for (mod in 1:nrow(table(moduleColors))){

modules = names(table(moduleColors))[mod]

probes = colnames(datExpr0)

inModule = (moduleColors == modules)

modGenes = probes[inModule]

write.table(modGenes, file =paste0("module_",modules,".txt"),sep="\t",row.names=F,col.names=F,quote=F)

}

geneSigFilter=0.5

moduleSigFilter=0.8

datMM=cbind(geneModuleMembership, geneTraitSignificance)

datMM=datMM[abs(datMM[,ncol(datMM)])>geneSigFilter,]

for(mmi in colnames(datMM)[1:(ncol(datMM)-2)]){

dataMM2=datMM[abs(datMM[,mmi])>moduleSigFilter,]

write.table(row.names(dataMM2), file =paste0("hubGenes_",mmi,".txt"),sep="\t",row.names=F,col.names=F,quote=F)

}

4. model.R：Multi-model Machine Learning Construction and Feature Selection

This script aims to construct and compare multiple machine learning classification models based on gene expression data. Four algorithms are employed: Random Forest (RF), Support Vector Machine (SVM), XGBoost, and Generalized Linear Model (GLM), with model performance evaluated via five-fold cross-validation. The DALEX framework is utilized for model interpretation, ROC curves are plotted to compare discriminatory power, and feature importance rankings are calculated for each model. The script outputs lists of top-contributing genes for each model (e.g., importanceGene.XGB.txt) to identify potential biomarkers.

library(caret)

library(DALEX)

library(ggplot2)

library(randomForest)

library(kernlab)

library(xgboost)

library(pROC)

set.seed(123)

inputFile="normalize.txt"

geneFile="interGenes.txt"

data=read.table(inputFile, header=T, sep="\t", check.names=F, row.names=1)

geneRT=read.table(geneFile, header=F, sep="\t", check.names=F)

data=data[as.vector(geneRT[,1]),]

row.names(data)=gsub("-", "_", row.names(data))

data=t(data)

group=gsub("(.*)\\_(.*)", "\\2", row.names(data))

data=as.data.frame(data)

data$Type=group

inTrain<-createDataPartition(y=data$Type, p=0.7, list=F)

train<-data[inTrain,]

test<-data[-inTrain,]

control=trainControl(method="repeatedcv", number=5, savePredictions=TRUE)

mod_rf = train(Type ~ ., data = train, method='rf', trControl = control)

mod_svm=train(Type ~., data = train, method = "svmRadial", prob.model=TRUE, trControl=control)

mod_xgb=train(Type ~., data = train, method = "xgbDART", trControl=control)

mod_glm=train(Type ~., data = train, method = "glm", family="binomial", trControl=control)

p_fun=function(object, newdata){

predict(object, newdata=newdata, type="prob")[,2]

}

yTest=ifelse(test$Type=="Control", 0, 1)

explainer_rf=explain(mod_rf, label = "RF",

data = test, y = yTest,

predict_function = p_fun,

verbose = FALSE)

mp_rf=model_performance(explainer_rf)

explainer_svm=explain(mod_svm, label = "SVM",

data = test, y = yTest,

predict_function = p_fun,

verbose = FALSE)

mp_svm=model_performance(explainer_svm)

explainer_xgb=explain(mod_xgb, label = "XGB",

data = test, y = yTest,

predict_function = p_fun,

verbose = FALSE)

mp_xgb=model_performance(explainer_xgb)

explainer_glm=explain(mod_glm, label = "GLM",

data = test, y = yTest,

predict_function = p_fun,

verbose = FALSE)

mp_glm=model_performance(explainer_glm)

pdf(file="residual.pdf", width=6, height=6)

p1 <- plot(mp_rf, mp_svm, mp_xgb, mp_glm)

print(p1)

dev.off()

pdf(file="boxplot.pdf", width=6, height=6)

p2 <- plot(mp_rf, mp_svm, mp_xgb, mp_glm, geom = "boxplot")

print(p2)

dev.off()

pred1=predict(mod_rf, newdata=test, type="prob")

pred2=predict(mod_svm, newdata=test, type="prob")

pred3=predict(mod_xgb, newdata=test, type="prob")

pred4=predict(mod_glm, newdata=test, type="prob")

roc1=roc(yTest, as.numeric(pred1[,2]))

roc2=roc(yTest, as.numeric(pred2[,2]))

roc3=roc(yTest, as.numeric(pred3[,2]))

roc4=roc(yTest, as.numeric(pred4[,2]))

pdf(file="ROC.pdf", width=5, height=5)

plot(roc1, print.auc=F, legacy.axes=T, main="", col="red")

plot(roc2, print.auc=F, legacy.axes=T, main="", col="blue", add=T)

plot(roc3, print.auc=F, legacy.axes=T, main="", col="green", add=T)

plot(roc4, print.auc=F, legacy.axes=T, main="", col="yellow", add=T)

legend('bottomright',

c(paste0('RF: ',sprintf("%.03f",roc1$auc)),

paste0('SVM: ',sprintf("%.03f",roc2$auc)),

paste0('XGB: ',sprintf("%.03f",roc3$auc)),

paste0('GLM: ',sprintf("%.03f",roc4$auc))),

col=c("red","blue","green","yellow"), lwd=2, bty = 'n')

dev.off()

importance_rf<-variable_importance(

explainer_rf,

loss_function = loss_root_mean_square

)

importance_svm<-variable_importance(

explainer_svm,

loss_function = loss_root_mean_square

)

importance_glm<-variable_importance(

explainer_glm,

loss_function = loss_root_mean_square

)

importance_xgb<-variable_importance(

explainer_xgb,

loss_function = loss_root_mean_square

)

pdf(file="importance.pdf", width=7, height=10)

plot(importance_rf[c(1,(ncol(data)-8):(ncol(data)+1)),],

importance_svm[c(1,(ncol(data)-8):(ncol(data)+1)),],

importance_xgb[c(1,(ncol(data)-8):(ncol(data)+1)),],

importance_glm[c(1,(ncol(data)-8):(ncol(data)+1)),])

dev.off()

geneNum=5

write.table(importance_rf[(ncol(data)-geneNum+2):(ncol(data)+1),], file="importanceGene.RF.txt", sep="\t", quote=F, row.names=F)

write.table(importance_svm[(ncol(data)-geneNum+2):(ncol(data)+1),], file="importanceGene.SVM.txt", sep="\t", quote=F, row.names=F)

write.table(importance_xgb[(ncol(data)-geneNum+2):(ncol(data)+1),], file="importanceGene.XGB.txt", sep="\t", quote=F, row.names=F)

write.table(importance_glm[(ncol(data)-geneNum+2):(ncol(data)+1),], file="importanceGene.GLM.txt", sep="\t", quote=F, row.names=F)

5. testROC.R: Independent Validation Set Model Performance Evaluation

This script conducts external validation of the constructed models on an independent test dataset. Based on input gene lists deemed important by specific models (e.g., importanceGene.XGB.txt output from model.R), it retrains the corresponding model on an independently standardized test set (test.normalize.txt) and evaluates its classification performance.

library(caret)

library(DALEX)

library(ggplot2)

library(randomForest)

library(kernlab)

library(xgboost)

library(pROC)

set.seed(123)

inputFile="test.normalize.txt"

geneFile="importanceGene.XGB.txt"

data=read.table(inputFile, header=T, sep="\t", check.names=F, row.names=1)

row.names(data)=gsub("-", "_", row.names(data))

geneRT=read.table(geneFile, header=T, sep="\t", check.names=F)

data=data[as.vector(geneRT[,1]),]

data=t(data)

group=gsub("(.*)\\_(.*)", "\\2", row.names(data))

data=as.data.frame(data)

data$Type=group

inTrain<-createDataPartition(y=data$Type, p=0.7, list=F)

train<-data[inTrain,]

test<-data[-inTrain,]

control=trainControl(method="repeatedcv", number=5, savePredictions=TRUE)

if(geneFile=="importanceGene.RF.txt"){

model=train(Type ~ ., data = train, method='rf', trControl = control)

}else if(geneFile=="importanceGene.SVM.txt"){

model=train(Type ~., data = train, method = "svmRadial", prob.model=TRUE, trControl=control)

}else if(geneFile=="importanceGene.XGB.txt"){

model=train(Type ~., data = train, method = "xgbDART", trControl=control)

}else if(geneFile=="importanceGene.GLM.txt"){

model=train(Type ~., data = train, method = "glm", family="binomial", trControl=control)

}

yTest=ifelse(test$Type=="Control", 0, 1)

pred1=predict(model, newdata=test, type="prob")

roc1=roc(yTest, as.numeric(pred1[,2]))

ci1=ci.auc(roc1, method="bootstrap")

ciVec=as.numeric(ci1)

pdf(file="ROC.pdf", width=5, height=5)

plot(roc1, print.auc=T, legacy.axes=T, main="", col="red")

text(0.39, 0.43, paste0("95% CI: ",sprintf("%.03f",ciVec[1]),"-",sprintf("%.03f",ciVec[3])), col="red")

dev.off()
