# Supplementary material for: Identification and validation of novel platelet-related genes in ulcerative colitis using bioinformatics and machine learning strategies
Source: PeerJ. 2026 Aug 25;14:e21655. doi: 10.7717/peerj.21655 (PMC13525761; doi:10.7717/peerj.21655)
Supplement: Supplementary file 5 [file peerj-14-21655-s002.docx]

**1**. **RAW data of body weight in DSS-induced colitis model**

|  | Control group weight (g) | | | | | DSS group weight (g) | | | | |
| --- | --- | --- | --- | --- | --- | --- | --- | --- | --- | --- |
| Day0 | 23.02 | 21.26 | 25.23 | 23.25 | 22.12 | 22.73 | 22.56 | 22.44 | 25 | 22.63 |
| Day1 | 23.14 | 21.52 | 25.19 | 23.01 | 22.41 | 23.44 | 22.87 | 22.81 | 25.1 | 22.21 |
| Day2 | 23.09 | 21.89 | 25.85 | 22.96 | 22.38 | 23.95 | 22.85 | 23.22 | 25 | 22.42 |
| Day3 | 23.9 | 22.17 | 25.47 | 23.45 | 22.44 | 23.68 | 22.65 | 22.94 | 24.3 | 21.98 |
| Day4 | 23.77 | 22.06 | 25.82 | 23.32 | 23.1 | 23 | 23.51 | 23.89 | 24.09 | 21.2 |
| Day5 | 23.61 | 22.13 | 26.81 | 23.72 | 23.25 | 22.3 | 22.64 | 22.06 | 23.94 | 20.87 |
| Day6 | 24.52 | 22.05 | 26.33 | 23.81 | 23.13 | 21.4 | 20.88 | 21.58 | 22.9 | 19.71 |
| Day7 | 24.94 | 22.64 | 26.79 | 23.76 | 23.64 | 21 | 19.73 | 19.43 | 23 | 18.8 |
| Day8 | 24.33 | 22.5 | 26.6 | 24.17 | 23.52 | 20.5 | 19.7 | 19.41 | 22.5 | 18.62 |

2. **RAW data of weight change in DSS-induced colitis model**

|  | Control group weight change (%) | | | | | DSS group weight change (%) | | | | |
| --- | --- | --- | --- | --- | --- | --- | --- | --- | --- | --- |
| Day0 | 0 | 0 | 0 | 0 | 0 | 0 | 0 | 0 | 0 | 0 |
| Day1 | 0.521286 | 1.222954 | -0.15854 | -1.03226 | 1.311031 | 3.123625 | 1.374113 | 1.648841 | 0.4 | -1.85594 |
| Day2 | 0.304083 | 2.963311 | 2.457392 | -1.24731 | 1.175407 | 5.367356 | 1.285461 | 3.475936 | 0 | -0.92797 |
| Day3 | 3.822763 | 4.280339 | 0.951249 | 0.860215 | 1.446655 | 4.179498 | 0.398936 | 2.228164 | -2.8 | -2.87229 |
| Day4 | 3.258036 | 3.762935 | 2.338486 | 0.301075 | 4.43038 | 1.187857 | 4.210993 | 6.461676 | -3.64 | -6.31905 |
| Day5 | 2.562989 | 4.092192 | 6.262386 | 2.021505 | 5.108499 | -1.89177 | 0.35461 | -1.6934 | -4.24 | -7.77729 |
| Day6 | 6.516073 | 3.715898 | 4.359889 | 2.408602 | 4.566004 | -5.8513 | -7.44681 | -3.83244 | -8.4 | -12.9032 |
| Day7 | 8.340573 | 6.491063 | 6.183115 | 2.193548 | 6.871609 | -7.61109 | -12.5443 | -13.4135 | -8 | -16.9244 |
| Day8 | 5.690704 | 5.832549 | 5.430044 | 3.956989 | 6.329114 | -9.81082 | -12.6773 | -13.5027 | -10 | -17.7198 |

**3.RAW data of DAI score in DSS-induced colitis model**

| Weight loss score | Days |  | |  | |  | |  | |  | |  |  |  | |  | |  |
| --- | --- | --- | --- | --- | --- | --- | --- | --- | --- | --- | --- | --- | --- | --- | --- | --- | --- | --- |
|  | Day0 | 0 | | 0 | | 0 | | 0 | | 0 | | 0 | 0 | 0 | | 0 | | 0 |
|  | Day1 | 0 | | 0 | | 0 | | 0 | | 0 | | 0 | 0 | 0 | | 0 | | 0 |
|  | Day2 | 0 | | 0 | | 0 | | 0 | | 0 | | 0 | 0 | 0 | | 0 | | 0 |
|  | Day3 | 0 | | 0 | | 0 | | 0 | | 0 | | 0 | 0 | 0 | | 0 | | 0 |
|  | Day4 | 0 | | 0 | | 0 | | 0 | | 0 | | 0 | 0 | 0 | | 0 | | 0 |
|  | Day5 | 0 | | 0 | | 0 | | 0 | | 0 | | 1 | 1 | 0 | | 0 | | 0 |
|  | Day6 | 0 | | 0 | | 0 | | 0 | | 0 | | 1 | 3 | 0 | | 0 | | 1 |
|  | Day7 | 0 | | 0 | | 0 | | 0 | | 0 | | 2 | 2 | 1 | | 1 | | 1 |
|  | Day8 | 0 | | 0 | | 0 | | 0 | | 0 | | 2 | 2 | 2 | | 3 | | 2 |
| Stool consistency and blood stool score | Days |  | |  | |  | |  | |  | |  |  |  | |  | |  |
|  | Day0 | 0 | | 0 | | 0 | | 0 | | 0 | | 0 | 0 | 0 | | 0 | | 0 |
|  | Day1 | 0 | | 0 | | 0 | | 0 | | 0 | | 0.5 | 0 | 0 | | 0 | | 0.5 |
|  | Day2 | 0 | | 0 | | 0 | | 0 | | 0 | | 0 | 0 | 0 | | 0 | | 0 |
|  | Day3 | 0 | | 0 | | 0 | | 0 | | 0 | | 0 | 0 | 0 | | 0 | | 0 |
|  | Day4 | 0 | 0 | | 0 | | 0 | | 0 | | 0.5 | 0 | 0 | | 0 | | 0 | |
|  | Day5 | 0 | 0 | | 0 | | 0 | | 0 | | 1.5 | 0.5 | 2.5 | | 1.5 | | 0.5 | |
|  | Day6 | 0 | 0 | | 0 | | 0 | | 0 | | 1.5 | 0.5 | 1 | | 0.5 | | 2 | |
|  | Day7 | 0 | 0 | | 0 | | 0 | | 0 | | 2 | 1.5 | 0.5 | | 1 | | 2.5 | |
|  | Day8 | 0 | 0 | | 0 | | 0 | | 0 | | 5 | 4 | 3 | | 5 | | 5 | |
|  |  |  |  | |  | |  | |  | |  |  |  | |  | |  | |
| Body posture score | Days |  |  | |  | |  | |  | |  |  |  | |  | |  | |
|  | Day0 | 0 | 0 | | 0 | | 0 | | 0 | | 0 | 0 | 0 | | 0 | | 0 | |
|  | Day1 | 0 | 0 | | 0 | | 0 | | 0 | | 0 | 0 | 0 | | 0 | | 0 | |
|  | Day2 | 0 | 0 | | 0 | | 0 | | 0 | | 0 | 0 | 0 | | 0 | | 0 | |
|  | Day3 | 0 | 0 | | 0 | | 0 | | 0 | | 0 | 0 | 0 | | 0 | | 0 | |
|  | Day4 | 0 | 0 | | 0 | | 0 | | 0 | | 0 | 0 | 0 | | 0 | | 0 | |
|  | Day5 | 0 | 0 | | 0 | | 0 | | 0 | | 0 | 0 | 0 | | 0 | | 0 | |
|  | Day6 | 0 | 0 | | 0 | | 0 | | 0 | | 0.5 | 1 | 0.5 | | 1 | | 1 | |
|  | Day7 | 0 | 0 | | 0 | | 0 | | 0 | | 1 | 1 | 1 | | 1 | | 1 | |
|  | Day8 | 0 | 0 | | 0 | | 0 | | 0 | | 2 | 2 | 2 | | 1 | | 1 | |
|  |  |  |  | |  | |  | |  | |  |  |  | |  | |  | |
| Total disease activity index (DAI) score. | Day0 |  |  | |  | |  | |  | |  |  |  | |  | |  | |
|  | Day1 | 0 | 0 | | 0 | | 0 | | 0 | | 0 | 0 | 0 | | 0 | | 0 | |
|  | Day2 | 0 | 0 | | 0 | | 0 | | 0 | | 0.5 | 0 | 0 | | 0 | | 0.5 | |
|  | Day3 | 0 | 0 | | 0 | | 0 | | 0 | | 0 | 0 | 0 | | 0 | | 0 | |
|  | Day4 | 0 | 0 | | 0 | | 0 | | 0 | | 0 | 0 | 0 | | 0 | | 0 | |
|  | Day5 | 0 | 0 | | 0 | | 0 | | 0 | | 1.5 | 1 | 0 | | 0 | | 0 | |
|  | Day6 | 0 | 0 | | 0 | | 0 | | 0 | | 2.5 | 3.5 | 2.5 | | 1.5 | | 1.5 | |
|  | Day7 | 0 | 0 | | 0 | | 0 | | 0 | | 4 | 3.5 | 2.5 | | 2.5 | | 4 | |
|  | Day8 | 0 | 0 | | 0 | | 0 | | 0 | | 5 | 4.5 | 3.5 | | 5 | | 5.5 | |
|  | Day0 | 0 | 0 | | 0 | | 0 | | 0 | | 7 | 6 | 5 | | 6 | | 6 | |

**4. RAW data of colon length in DSS-induced colitis model**

| **Colon length/cm** |  |  |
| --- | --- | --- |
|  | WT | DSS |
|  | 6.6 | 5.7 |
|  | 6.2 | 5.1 |
|  | 5.3 | 4.4 |
|  | 7.0 | 4.8 |
|  | 6.7 | 5.11 |

**5. RAW data of histology score in DSS-induced colitis model**

|  | wt1 | wt2 | wt3 | wt4 | dss1 | dss2 | dss3 | dss4 |
| --- | --- | --- | --- | --- | --- | --- | --- | --- |
| Ulcers (number) | 0 | 0 | 0 | 0 | 1 | 0 | 0 | 1 |
| Epithelial cell changes | 1 | 0 | 0 | 0 | 3 | 3 | 3 | 3 |
| Inflammatory infiltration | 0 | 0 | 0 | 1 | 4 | 4 | 4 | 4 |
| Final score | 1 | 0 | 0 | 1 | 8 | 7 | 7 | 8 |

**6. RAW data of qRT-PCR in DSS-induced colitis model**

| QPCR/MOUSE | | |
| --- | --- | --- |
| timp1 | WT | DSS |
|  | 1 | 602.604 |
|  | 2.956 | 391.0261 |
|  | 9 5129 | 177.8139 |
|  | 1 926 | 187.7446 |
|  | | |
| Trim22 | WT | DSS |
|  | 1 | 4.2168 |
|  | 0.4907 | 3.6448 |
|  | 0.7699 | 2.4945 |
|  | 0.1354 | 1.193 |
|  | | |
| Rac1/2/3 | WT | DSS |
|  | 1 | 70.6974 |
|  | 2.956 | 253.3705 |
|  | 0.1553 | 0.6188 |
|  | 1.9519 | 6.1268 |

**7. RAW data of qRT-PCR in clinical UC blood PBMC samples**

| QPCR/HUMAN | | | | | | | |
| --- | --- | --- | --- | --- | --- | --- | --- |
| RAC2 | |  | TIMP1 | |  | TRIM22 | |
| HC | UC |  | HC | UC |  | HC | UC |
| 3.45 | 11.0869 |  | 12.6798 | 17.5188 |  | 4.49665 | 18.4784 |
| 3.28422 | 10.9341 |  | 15.1221 | 13.1641 |  | 1.4048 | 8.26715 |
| 6.88819 | 6.64961 |  | 21.8758 | 18.3956 |  | 2.09786 | 4.30437 |
| 3.09554 | 4.11779 |  | 4.97105 | 22.5443 |  | 3.06836 | 0.64543 |
| 5.17358 | 6.03653 |  | 3.85339 | 23.0974 |  | 7.04675 | 1.51404 |
| 5.44969 | 10.502 |  | 10.9162 | 14.6377 |  | 1.23656 | 17.9057 |
| 4.06104 | 15.527 |  | 24.8093 | 10.3294 |  | 4.7045 | 15.965 |
| 2.0611 | 1.02616 |  | 17.8033 | 11.3023 |  | 10.0073 | 13.7594 |
| 1.04228 | 3.55972 |  | 4.95893 | 6.3387 |  | 4.57481 | 16.4573 |
| 3.06665 | 5.46163 |  | 12.823 | 20.3441 |  | 10.1125 | 6.70126 |
| 1.2376 | 7.42454 |  | 17.532 | 11.7907 |  | 7.573 | 9.44751 |
| 2.36083 | 13.9568 |  | 6.91243 | 25.8981 |  | 5.97718 | 7.77034 |
| 12.7483 | 14.3243 |  | 7.5461 | 11.8585 |  | 10.1905 | 2.53713 |
| 5.72715 | 6.24377 |  | 12.3974 | 12.0264 |  | 3.90406 | 8.87749 |
| 7.93176 | 9.25782 |  | 7.59494 | 11.6469 |  | 2.67124 | 2.31312 |
| 2.67571 | 7.57219 |  | 10.7183 | 13.0067 |  | 6.2889 | 8.51606 |
| 4.76134 | 8.93758 |  | 7.57882 | 8.94591 |  | 1.96932 | 8.26729 |
| 12.7483 | 4.72994 |  | 7.62702 | 21.8082 |  | 10.7045 | 12.5959 |
| 14.5688 | 4.6959 |  | 8.15809 | 27.0201 |  | 1.6568 | 9.60471 |
| 3.22064 | 3.77455 |  | 8.35672 | 12.4169 |  | 4.71346 | 1.42746 |
| 5.62481 | 6.65651 |  | 7.79365 | 26.4249 |  | 1.56273 | 8.04671 |
| 12.5938 | 9.1834 |  | 13.3587 | 24.7235 |  | 5.50927 | 7.77351 |
| 6.46868 | 3.35382 |  | 7.91931 | 11.9502 |  | 1.50714 | 9.16726 |
| 2.66917 | 2.71881 |  | 11.3544 | 12.4708 |  | 1.69599 | 14.1358 |
| 6.59378 | 4.44846 |  | 7.42983 | 19.772 |  | 0.96263 | 2.4658 |
| 3.03407 | 6.1494 |  | 6.63535 | 11.6681 |  | 1.69989 | 12.624 |
| 9.53374 | 9.3461 |  | 10.5236 | 12.3898 |  | 4.8651 | 8.35297 |
| 7.74179 | 6.21112 |  | 7.17574 | 24.5546 |  | 3.42655 | 8.33896 |
| 2.84305 | 14.2045 |  | 5.42983 | 12.4088 |  | 1.52504 | 8.4431 |
| 10.463 | 13.7136 |  | 7.42983 | 17.9085 |  | 4.55402 | 7.50681 |
| 2.45016 | 4.59076 |  | 7.51642 | 13.0958 |  | 2.19185 | 7.94354 |
| 2.72715 | 13.9893 |  | 6.97553 | 12.689 |  | 0.88134 | 2.17001 |
| 12.5393 | 14.464 |  | 7.27843 | 11.3277 |  | 2.64424 | 2.37695 |
| 3.08485 | 14.6104 |  | 13.4429 | 19.9992 |  | 1.34894 | 4.0973 |
| 4.18996 | 5.92044 |  | 7.68046 | 25.689 |  | 1.70059 | 8.85143 |
| 3.0382 | 2.65651 |  | 6.95827 | 12.9597 |  | 4.65216 | 7.63916 |
| 2.78203 | 13.8393 |  | 7.51642 | 7.97392 |  | 4.97234 | 9.28313 |
| 6.52739 | 4.16968 |  | 15.2256 | 21.9291 |  | 5.34894 | 8.59884 |
| 2.71406 | 10.5866 |  | 12.4705 | 8.83762 |  | 1.74786 | 8.85265 |
| 1.70778 | 3.94333 |  | 17.4507 | 24.3996 |  | 1.33468 | 3.30396 |
| 6.0243 | 4.33154 |  | 22.0285 | 11.482 |  | 1 | 7.9123 |
| 2.56883 | 13.9248 |  | 7.51642 | 11.5379 |  | 4.04665 | 8.08785 |
| 3.0461 | 3.98231 |  | 6.85799 | 6.95023 |  | 2.54018 | 2.66141 |
| 6.64333 | 3.87537 |  | 19.7783 | 12.1844 |  | 4.68463 | 7.91719 |
| 12.4136 | 4.89978 |  | 7.28338 | 12.0647 |  | 5.79725 | 7.70673 |
| 3.18113 | 4.02073 |  | 7.51866 | 7.4196 |  | 7.9098 | 7.6824 |
| 2.42594 | 11.9893 |  | 11.3805 | 12.4795 |  | 1.17372 | 7.53057 |
| 12.5505 | 10.0271 |  | 7.07757 | 6.23523 |  | 0.97808 | 5.17293 |
| 2.4379 | 16.8998 |  | 6.06916 | 12.1936 |  | 6.6092 | 8.02992 |
| 2.71406 | 3.83374 |  | 7.3019 | 7.3305 |  | 6.73568 | 1.01642 |
| 4.2376 | 4.2946 |  | 19.7013 | 12.2274 |  | 9.70133 | 9.4544 |
| 3.36083 | 14.5462 |  | 9.93315 | 15.3422 |  | 9.93315 | 10.3845 |
| 6.7483 | 14.3382 |  | 7.36768 | 12.8119 |  | 9.36768 | 6.21624 |
| 13.7271 | 13.7441 |  | 9.83634 | 9.47366 |  | 10.8363 | 10.5765 |
| 2.93176 | 4.28745 |  | 12.4939 | 12.9161 |  | 9.49389 | 10.5252 |
| 13.6757 | 3.63134 |  | 9.24827 | 8.2881 |  | 11.2483 | 9.88282 |
| 13.7613 | 13.9376 |  | 8.83717 | 12.605 |  | 9.83717 | 9.55163 |
| 10.7483 | 4.00162 |  | 9.49665 | 6.28558 |  | 9.49665 | 13.9635 |
| 11.5688 | 13.9248 |  | 11.4048 | 12.0943 |  | 3.4048 | 10.2666 |
| 5.46 | 3.88134 |  | 6.666 | 19.0599 |  | 3.233 | 14.6061 |
