# Supplementary material for: Identification and validation of novel platelet-related genes in ulcerative colitis using bioinformatics and machine learning strategies
Source: PeerJ. 2026 Aug 25;14:e21655. doi: 10.7717/peerj.21655 (PMC13525761; doi:10.7717/peerj.21655)
Supplement: Supplementary file 6 [file peerj-14-21655-s005.docx]

**Table S1 Details of the sample information of the datasets.**

| Dataset | Platform | Data modality | Sample source | Organism | Control samples | UC samples | Role in study |
| --- | --- | --- | --- | --- | --- | --- | --- |
| GSE11223 | GPL1708 | Bulk transcriptomic microarray | Tissue | Homo sapiens | 73 | 129 | Discovery/model-training cohort |
| GSE107499 | GPL15207 | Bulk transcriptomic microarray | Tissue | Homo sapiens | 44 | 75 | External tissue validation cohort |
| GSE92415 | GPL13158 | Bulk transcriptomic microarray | Tissue | Homo sapiens | 21 | 162 | External tissue validation cohort |
| GSE119600 | GPL10558 | Bulk transcriptomic microarray | Blood | Homo sapiens | 47 | 93 | External blood validation cohort |
| GSE3365 | GPL96 | Bulk transcriptomic microarray | Blood | Homo sapiens | 42 | 26 | External blood validation cohort |
| GSE94648 | GPL19109 | Bulk transcriptomic microarray | Blood | Homo sapiens | 22 | 25 | External blood validation cohort |
| GSE214695 | GPL18573 | Single-cell RNA-seq | Tissue | Homo sapiens | 6 | 6 | Single-cell expression localization |
