# Supplementary material for: Identification and validation of novel platelet-related genes in ulcerative colitis using bioinformatics and machine learning strategies
Source: PeerJ. 2026 Aug 25;14:e21655. doi: 10.7717/peerj.21655 (PMC13525761; doi:10.7717/peerj.21655)
Supplement: Supplementary file 7 [file peerj-14-21655-s007.docx]

Table S3. Sequences of primers used for qRT-PCR.

| **Species** | **Genes** | **Sequences** |
| --- | --- | --- |
| Human | β-actin | Forward: 5’- TGTGACGTGGACATCCGCAAAG -3’  Reverse: 5’- TGGAAGGTGGACAGCGAGGC-3’ |
| Human | *TRIM22* | Forward: 5’-AAACATTCCGCATAAACGA-3’  Reverse: 5’-ATCCAGCACATTCACCTCAC-3’ |
| Human | *RAC2* | Forward: 5-’CAATGTGATGGTGGACAGCAAG-3’  Reverse: 5’ -GAGGGAGAAGCAGATGAGGAAGA-3’ |
| Human | *TIMP1* | Forward: 5’-CTGCGGATACTTCCACAGGTC-3’  Reverse: 5’- CTCACAGCCAACAGTGTAGGTCTT-3’ |
| Mouse | *Trim22* | Forward: 5’- CCGCCTGGAAGATCGAGAG-3’  Reverse: 5’- CTGCCAGGTTATCCAGCACA-3’ |
| Mouse | *Rac2* | Forward: 5’- GACAGTAAGCCGGTGAACCTG-3’  Reverse: 5’- CTGACTAGCGAGAAGCAGATG-3’ |
| Mouse | *Timp1* | Forward: 5’- TCCAAGGCTCTGAAAAGGGC-3’  Reverse: 5’- ATTCAGGCTATCTGGGACCG-3’ |
| Mouse | *Hprt* | Forward: 5’-GTCCCAGCGTCGTGATTAGC-3’  Reverse: 5’- TGGCCTCCCATCTCCTTCA-3’ |
